# Dopamine D1 receptor signalling in dyskinetic Parkinsonian rats revealed by fiber photometry using FRET-based biosensors

**DOI:** 10.1038/s41598-020-71121-8

**Published:** 2020-09-02

**Authors:** Jace Jones-Tabah, Hanan Mohammad, Shadi Hadj-Youssef, Lucy E. H. Kim, Ryan D. Martin, Faïza Benaliouad, Jason C. Tanny, Paul B. S. Clarke, Terence E. Hébert

**Affiliations:** Department of Pharmacology and Therapeutics, McGill University, 3655 Promenade Sir-William-Osler, Room 1325, Montreal, QC H3G 1Y6 Canada

**Keywords:** Molecular neuroscience, Diseases of the nervous system, Parkinson's disease, Pharmacology, Receptor pharmacology

## Abstract

As with many G protein-coupled receptors (GPCRs), the signalling pathways regulated by the dopamine D1 receptor (D1R) are dynamic, cell type-specific, and can change in the face of disease or drug exposures. In striatal neurons, the D1R activates cAMP/protein kinase A (PKA) signalling. However, in Parkinson’s disease (PD), alterations in this pathway lead to functional upregulation of extracellular regulated kinases 1/2 (ERK1/2), contributing to l-DOPA-induced dyskinesia (LID). In order to detect D1R activation in vivo and to study the progressive dysregulation of D1R signalling in PD and LID, we developed ratiometric fiber-photometry with Förster resonance energy transfer (FRET) biosensors and optically detected PKA and ERK1/2 signalling in freely moving rats. We show that in Parkinsonian animals, D1R signalling through PKA and ERK1/2 is sensitized, but that following chronic treatment with l-DOPA, these pathways become partially desensitized while concurrently D1R activation leads to greater induction of dyskinesia.

## Introduction

G protein-coupled receptors (GPCRs) play pivotal roles in mediating neuronal communication in the brain. In fact, 90% of non-olfactory GPCRs are found in the brain^1^, where they regulate neuronal activity by engaging a variety of distal downstream effectors which include second messenger producing enzymes, ion channels, monomeric GTPases and protein kinases. Many GPCRs are pharmacologically targeted in the treatment of neurodegenerative and neuropsychiatric disease. Thus it is critical to understand how these receptors regulate intracellular signalling, and how this in turn regulates circuit function and ultimately, behavior. In cell culture models, these signalling pathways have been dissected extensively, through the use of genetically-encoded fluorescent and bioluminescent biosensors. Some of these biosensors have recently been used in the in vivo context, to link specific signalling patterns with behavioral outcomes^2–6^.

Many intracellular signalling biosensors utilize changes in Förster resonance energy transfer (FRET) between two fluorescent proteins^7^ to report levels or activity of second messengers, protein kinases, GTPases, post-translational modifications and protein–protein interactions^8–11^. These tools have been widely used to dissect signaling pathways in cultured cells and have recently begun to be applied in vivo*,* aided by recent technological developments in intravital imaging^10,12^, microendoscopy^3^, 2-photon microscopy^13–15^ and fluorescence lifetime measurement^16^. Here we report a ratiometric fiber-photometry approach for real-time recording of genetically-encoded FRET biosensors in freely moving rodents. We apply this approach to investigate alterations in striatal GPCR signalling in a rat model of Parkinson’s disease and l-DOPA induced dyskinesia.

The dopamine D1 receptor (D1R) is a Gα_s/olf_ coupled GPCR expressed throughout the forebrain. As for many GPCRs, several factors can impact D1R signalling, including the properties of specific ligands, the cellular context, interactions with signalling partners, and evolving disease processes. The D1R is particularly abundant in the striatum, where it is expressed on medium spiny GABAergic projection neurons that make up the “direct pathway” of the basal ganglia (dMSNs)^17,18^. In dMSNs, D1R activation increases neuronal excitability and promotes synaptic plasticity by stimulating cAMP production and protein kinase A (PKA)/DARPP-32-dependent phosphorylation of downstream targets^19,20^.

In Parkinson’s disease, degeneration of dopaminergic neurons in the substantia nigra results in striatal dopamine depletion, leading to a hypoactivity of dMSNs. In response to diminished synaptic dopamine levels, D1R signaling in dMSNs becomes rewired through alterations in receptor trafficking, upregulation of D1R-associated effectors including Gα_olf_, and formation of disease-specific receptor heteromers^21–25^. These adaptations effectively sensitize the D1R and increase its ability to activate multiple signaling pathways mediated by PKA and extracellular regulated kinases 1/2 (ERK1/2). Stimulation of sensitized D1Rs contributes to the development of a common adverse effect of Parkinson’s treatment termed l-DOPA-induced dyskinesia (LID)^26^. While numerous behavioral and biochemical studies have linked aberrations in D1R signalling to the development of LID, it was previously not possible to measure these intracellular biochemical signalling events repeatedly in the same animals. The ability to detect changes in signaling events in live animals in combination with behavioral analysis would provide a powerful approach to investigate potential treatments for PD and LID.

To study D1R-dependent signalling in the striatum, we expressed genetically-encoded FRET biosensors for PKA and ERK1/2 activity in the dorsal striatum of adult rats and recorded signalling dynamics in behaving animals using a newly developed ratiometric photometry technique. Using a 6-OHDA model of Parkinson’s disease, we show that D1R-mediated activation of PKA and ERK1/2 is at first enhanced by dopamine depletion, and partially attenuated by prolonged l-DOPA treatment. Despite this apparent reduction in disease-relevant signalling responses, dyskinesia increased in response to D1R stimulation, indicating that repeated l-DOPA exposure alters the relationship between signalling pathway activation and the dyskinesia phenotype. Our approach allowed us to monitor signalling in the same animals over the course of dopamine depletion and l-DOPA treatment, and can provide novel insights into how signalling processes shape behavioral outcomes over time.

## Results

### Cloning and evaluation of FRET-based protein kinase biosensors AKAR and EKAR in primary rat striatal neurons

**Figure 1:**
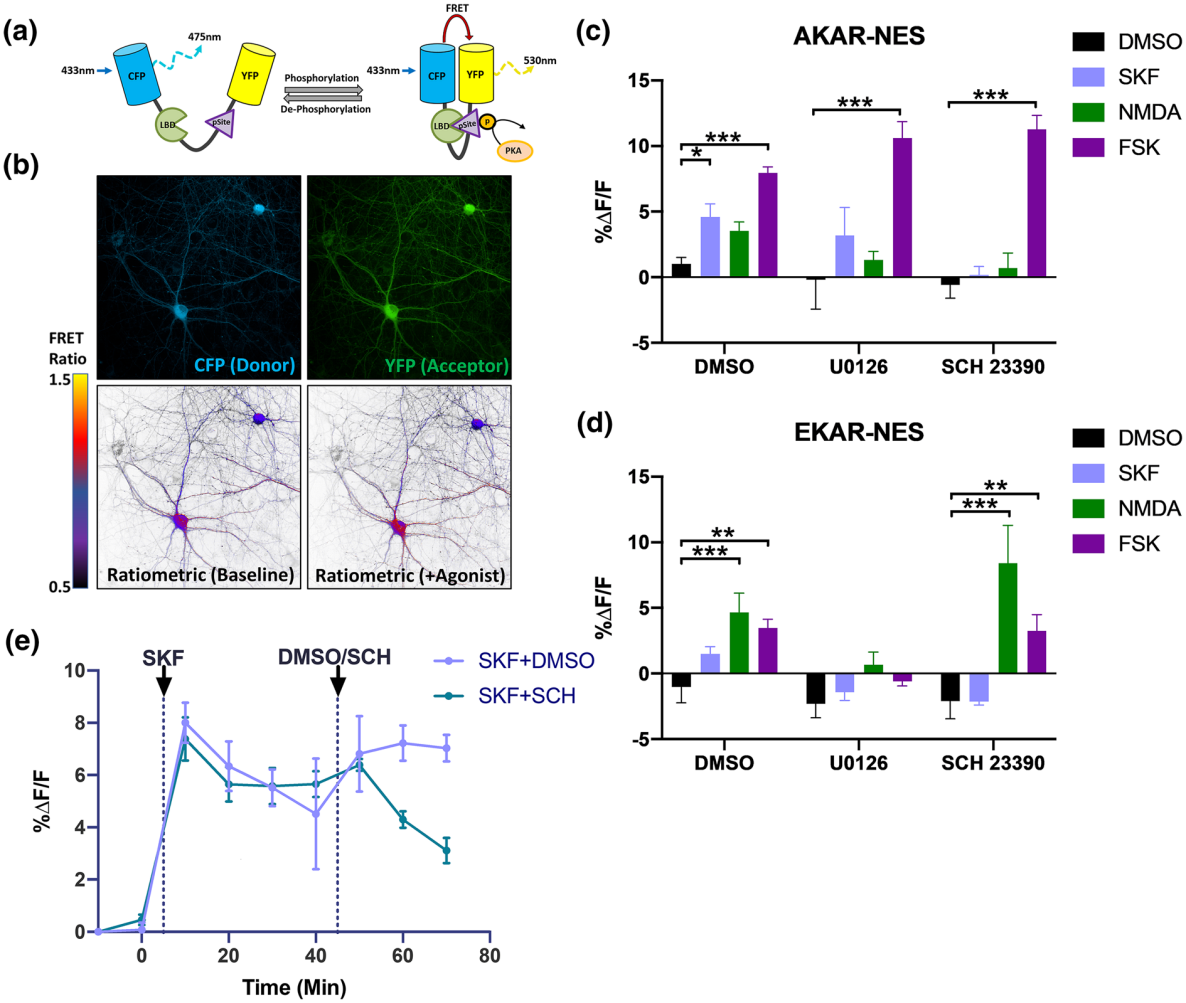
FRET-based protein kinase sensors AKAR and EKAR report signalling pathway activation in primary neuronal cultures. (**a**) Schematic depiction of the PKA biosensor, showing how the molecular rearrangement that results from PKA phosphorylation in turn leads to changes in FRET signal. The ERK1/2 biosensor EKAR works by an analogous mechanism. (**b**) Representative image of neurons transduced with AKAR showing the CFP donor (top left), YFP acceptor (top right), and ratiometric pseudo-images before and after treatment with the D1R agonist SKF 81297 (lower left and right, respectively). Ratiometric images were generated by plotting the calculated FRET ratio (YFP/CFP) at each image pixel according to the color scale shown. (**c**,**d**) Bar graphs depicting change in PKA (**c**) or ERK1/2 (**d**) biosensor activity in primary cultured neurons following a 5-min treatment with the indicated drugs and displayed as change in FRET relative to baseline FRET (%ΔF/F). (**e**) Time course of activation of PKA in response to D1R agonist SKF 81297, followed by reversal mediated by treatment with the D1R antagonist SCH 23390. Abbreviations and drug doses: DMSO = dimethyl-sulfoxide, SKF = SKF 81297 (1 µM), NMDA = *N*-methyl-d-aspartic acid (5 µM), FSK = forskolin (5 µM), U0126 (1 µM), SCH = SCH 23390 (1 µM). Data are displayed as mean ± SEM for 3–6 independent experiments. *p < 0.05, **p < 0.01, ***p < 0.001 vs. DMSO within each pre-treatment group (Bonferroni-corrected t-tests).

We set out to develop a platform for expressing and recording FRET-based biosensors in a way that was both simple to use and readily generalizable to other biosensors and animal models. We opted to use adeno-associated viral vectors (AAVs) to express previously published FRET-based reporters for PKA and ERK1/2, referred to as AKAR and EKAR respectively^9^. Variants of these biosensors have previously been used in vivo in applications such as microendoscopy^3,9^, 2-photon microscopy^14^, and fluorescence lifetime photometry^16^. These biosensors are scaffolded on substrates for protein kinase activity and contain phosphorylation-dependent interaction domains, such that the biosensor undergoes a conformational change upon phosphorylation, leading to an increase in resonance energy transfer (Fig. 1a). We subcloned AKAR and EKAR constructs into an AAV backbone containing a neuron-specific promoter and used AAV serotype 1 to transduce primary striatal neurons from rat brain (Fig. 1b–e). We confirmed neuron-specific expression by immunofluorescent labelling (Supplementary Fig. S1), and we also performed live-cell FRET imaging in 96 well plates using high-content confocal microscopy (Fig. 1b–e). In primary neurons, the AKAR-associated FRET ratio was increased by the addition of the D1R agonist SKF 81297 or the adenylyl cyclase activator, forskolin (Fig. 1c). The PKA response to SKF 81297 but not forskolin was inhibited by pre-treatment with the D1R antagonist SCH 23390, but not the MEK inhibitor U0126. Conversely, EKAR-associated FRET was increased by addition of NMDA or forskolin and was attenuated by pre-treatment with the MEK inhibitor U0126 (Fig. 1d). This pattern of results is consistent with previous studies indicating that D1R couples only weakly to ERK1/2 under normal physiological conditions^23^. Finally, the elevated PKA activity seen after acute SKF 81297 administration could be partially reversed by subsequent addition of a D1 antagonist, demonstrating that the FRET response can be reversed when signalling is attenuated (Fig. 1e).

### In vivo expression of FRET biosensors in rat striatum

**Figure 2:**
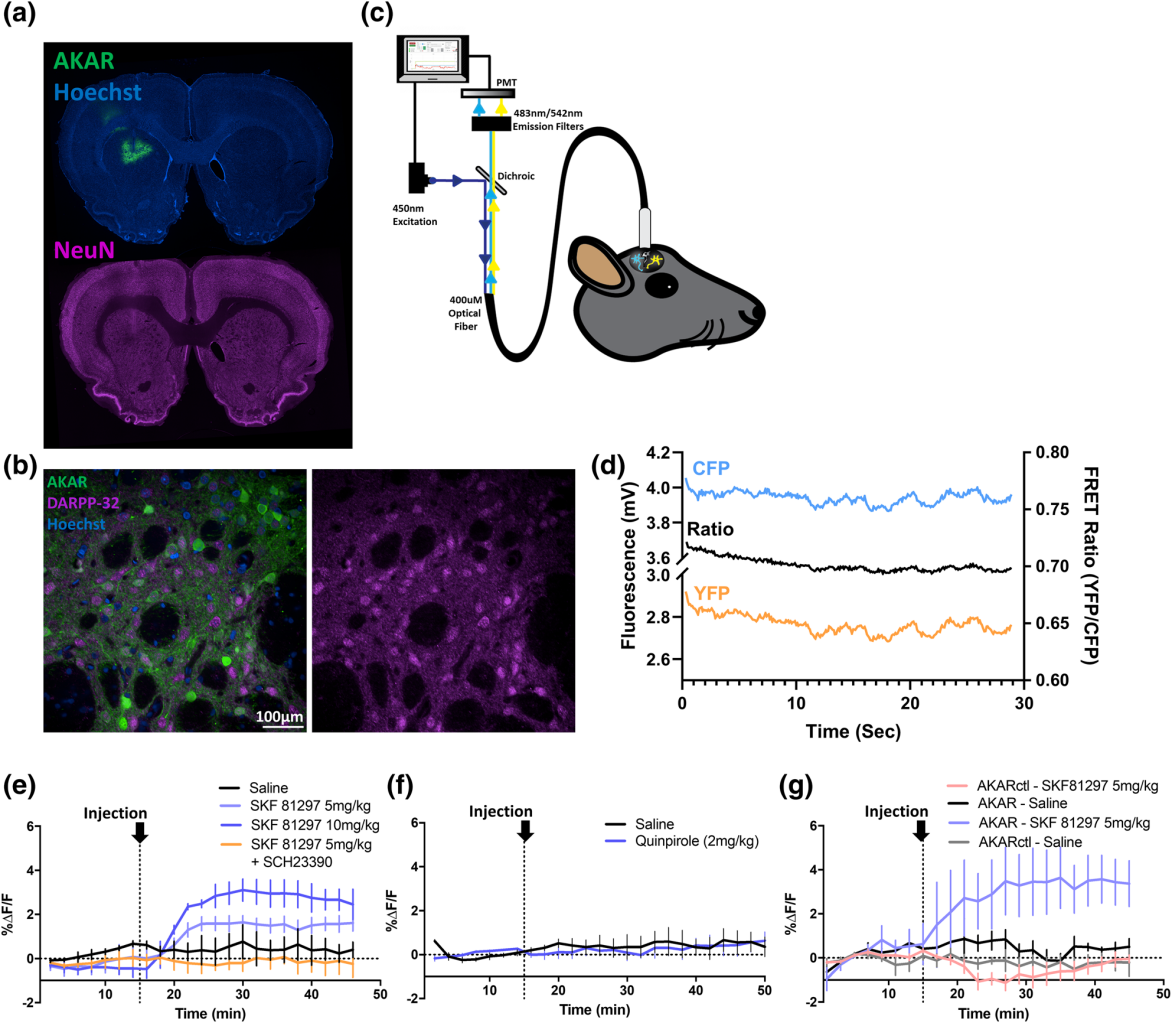
AAV-mediated expression of AKAR in rat striatum and photometry recording in anesthetized and freely moving rats. (**a**) Representative image showing expression of AKAR and staining for the neuronal marker NeuN in a coronal section containing the dorsal striatum (i.e. caudate-putamen) of an adult rat that was sacrificed 5 weeks following stereotaxic injection of AAV-AKAR. (**b**) Representative high-magnification image showing fluorescent DARPP-32 immunolabelling of fixed striatal tissue, together with AKAR expression. (**c**) Schematic representation of the ratiometric photometry system. (**d**) Exemplar trace showing CFP and YFP channels, and calculated FRET ratio, recorded for 30 s from AKAR-transduced rat dorsal striatum at 100 Hz sampling rate. The lower panels show photometry recordings obtained in rats maintained either under light isoflurane anesthesia (**e**,**f**) or freely moving (**g**), with FRET values averaged within consecutive 30-s time bins. (**e**) After 15 min of recording, rats were injected s.c. (vertical dotted line) with vehicle or the D1R agonist SKF 81297, with or without 30-min pre-treatment with the D1R antagonist SCH 23390. (**f**) Rats were injected with vehicle or the dopamine D2R agonist quinpirole. Responses were recorded for 30 s every 2 min. (**g**) Photometry recording performed in freely-moving rats with either AKAR or AKARctl, the latter being a negative control biosensor lacking a phosphorylation site. Rats were recorded at 2-min intervals, and were challenged with either vehicle or SKF 81297 (5 mg/kg s.c.), 15 min after the start of recording. For all experiments, individual rats were recorded under vehicle and drug conditions on consecutive days in a counterbalanced design. (**e**–**g**) These panels show the mean ± SEM (n = 3 rats), where the value for each rat is the mean FRET over a 30 s long 100-Hz recording such as is shown in (**d**). Refer to “Materials and methods” for full description of data processing and representation.

To express FRET biosensors in vivo*,* we injected AAV-AKAR (titer ~ 5 × 10^12^ viral genomes/ml) into the dorsal striatum of adult Sprague–Dawley rats. We then assessed expression 3 weeks later by immunofluorescence microscopy in fixed brain sections (Fig. 2a,b, Supplementary Fig. S2). Fluorescently labelled cells stained positive for NeuN and DARPP-32, but not the astrocytic marker GFAP, confirming that expression was restricted to neurons as previously been shown using this AAV vector^27^. In the rat striatum, 95% of neurons are medium spiny GABAergic projection neurons that express DARPP-32^28,29^. Of these, approximately half express D1R and contribute to the direct pathway projecting from the striatum to the substantia nigra reticulata (SNr), whereas the remainder express D2R and make up the indirect pathway projecting to the external globus pallidus (GPe)^30,31^. In a sagittal brain section, we observed fluorescently labelled axons projecting to both the GPe and SNr, suggesting transduction of neurons giving rise to either pathway (Supplementary Fig. S2d). Using dynorphin-A as a marker of dMSNs, we found that biosensor expression was localized to both dynorphin-positive and dynorphin-negative neurons, confirming the transduction of both striatal pathways (Supplementary Fig. S3).

### Development of CFP/YFP ratiometric photometry

Fiber photometry is a method for performing functional fluorescence recording in vivo and is compatible with many common behavioral tests^32–34^. We used a custom fiber-photometry system (Labeo Technologies, Montréal) to perform ratiometric FRET measurements through surgically implanted optical cannulae (schematically shown in Fig. 2c). Excitation and emission filters were selected based on the experimentally determined emission spectra of AKAR expressed in HEK 293 cells (Supplementary Fig. S4). Using excitation with a 450 nm laser-diode, we detected fluorescence signals corresponding to the FRET donor (CFP) and acceptor (YFP) from AAV-AKAR injected rats > threefold greater than background signals recorded from control rats which had a probe implanted but were not injected with virus (Supplementary Fig. S5). An example of the raw fluorescence trace and corresponding FRET ratio acquired at 100 Hz from a single rat expressing the AKAR sensor is shown in Fig. 2d. To minimize the potential for photobleaching during long duration recordings, laser illumination was applied as a train of discrete 30-s ON/OFF intervals (described further in Supplementary Fig. S6a). The mean fluorescence from each 30-s recording interval was then averaged in order to condense the data for analysis. Furthermore, illumination of brain tissue at 450 nm was found to produce approximately threefold greater autofluorescence in the CFP channel compared to the YFP channel, and this autofluorescence bleached upon extended illumination (Supplementary Fig. S6b). To remove this background autofluorescence, we recorded from two age-matched sham-injected control rats in each experiment and subtracted the average background signal corresponding to the time of recording for each FRET experiment (further described in “Materials and methods” and shown schematically in Supplementary Fig. S6c).

### D1R agonist dose-dependently activates PKA signalling in dorsal striatum

To test the ability of this in vivo system to detect agonist-induced effects on protein kinase signalling, we expressed AAV-AKAR in the dorsal striatum and recorded responses to systemically-administered D1 and D2 agonists (Fig. 2e–g). Photometric recording was initially performed with rats under light anesthesia in order to immobilize the animals and isolate drug-induced changes from behavior-associated fluctuations in striatal signaling that might occur in moving animals. Recording in anesthetized rats, we observed a rapid, and dose-dependent increase in PKA activation following subcutaneous injection of the D1R agonist SKF 81297 (Fig. 2e). In contrast, quinpirole, an agonist for D2 receptors, which are coupled to Gα_i_ rather than Gα_s_, elicited no response (Fig. 2f). The lack of negative FRET response to D2R activation in anesthetized rats is consistent with prior studies showing that PKA signalling in the indirect pathway is basally maintained at low levels and is not further reduced by D2R activation^35^. We next tested freely moving rats, recording FRET responses following s.c. injection of 5 mg/kg SKF 81297, a dose expected to stimulate locomotor activity and induce cFos in dMSNs^23,36^. We observed that while SKF 81297 treatment increased the FRET ratio in rats expressing the AKAR sensor, no effect was observed in rats expressing a control version of the biosensor in which the phosphorylation substrate site was mutated and does not undergo conformational change (Fig. 2g).

### D1R-dependent PKA activation is enhanced in a 6-OHDA lesion model of Parkinson’s disease

The histopathological hallmark of Parkinson’s disease (PD) is degeneration of nigral dopaminergic neurons, which in turn causes the main clinical symptoms of rigidity, tremor and akinesia^37^. Patients with PD do not typically present with motor deficits until they have lost at least 50% of dopamine neurons, and an even greater number of striatal dopamine terminals^38^. One potential explanation for this phenomenon is that compensation takes place through increased sensitivity of post-synaptic dopamine receptor signalling. In support of this hypothesis, lesion of the nigrostriatal dopamine pathway in animal models leads to behavioral and biochemical sensitization to D1R agonists^23,39^.

**Figure 3:**
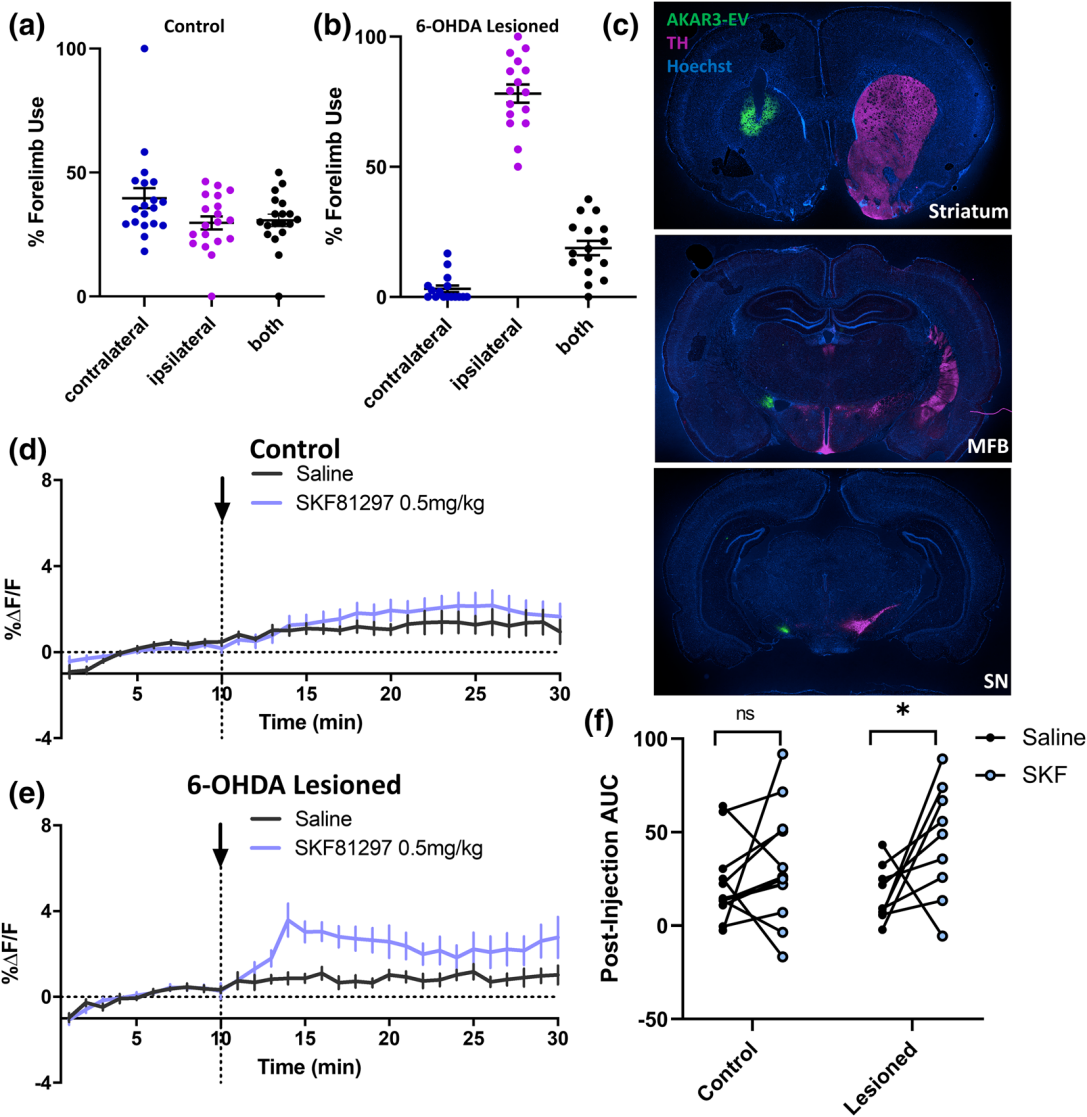
Using photometry to measure D1R-dependent PKA activation in intact and 6-OHDA lesioned hemi-Parkinsonian rats. (**a**,**b**) Cylinder test results for intact rats and 6-OHDA lesioned rats, respectively. Rats in both groups had been implanted with optic probes and expressed AKAR in the dorsal striatum. In the case of 6-OHDA injected animals AKAR was expressed ipsilateral to the lesion. Y-axes show the percentage of rears in which individual animals used the forelimb that was ipsilateral *vs.* contralateral to the AAV-injected hemisphere, or used both forelimbs at the same time. Tests in the 6-OHDA lesioned rats occurred 3 weeks following intra-striatal injection of AAV_1_-SynTet-AKAR and optical probe implantation. (**c**) Representative immunofluorescent images depicting AKAR expression (green) and optic-probe placement in the dorsal striatum in addition to expression of tyrosine hydroxylase (TH, shown in purple) in the striatum, medial forebrain bundle (MFB) and substantia nigra (SN). (**d**) FRET recordings performed on intact rats expressing AKAR and injected with either saline or the D1R agonist SKF 81297 (0.5 mg/kg s.c.) (n = 12). (**e**) FRET recordings performed on 6-OHDA lesioned rats expressing AKAR and injected with either saline or SKF81297 (0.5 mg/kg s.c.) (n = 9). For all experiments, rats were recorded under both saline and drug conditions in a counterbalanced design. (**f**) Area under the curve (AUC) analysis of (**d**) and (**e**). AUC was calculated relative to baseline following drug or saline injection. AUC values for Saline vs. SKF 81297 in each control and lesioned rats were by repeated measures t-tests, *p < 0.05.

We therefore sought to determine whether we could observe enhanced D1R-dependent PKA signalling in a rat model of PD. To this end, we used 6-hydroxydopamine (6-OHDA) to unilaterally lesion midbrain dopamine neurons^40^. A hemi-Parkinsonian phenotype was confirmed, first behaviorally using the cylinder test of forelimb use asymmetry (Fig. 3a,b), and subsequently by tyrosine hydroxylase (TH) immunostaining at the end of each experiment (Fig. 3c). We also verified that expression of AAV-AKAR, together with optic probe implantation in the striatum, did not itself alter performance in the cylinder test (Fig. 3a). We expressed AAV-AKAR in the striatum of the lesioned hemisphere and recordings were performed 4 weeks post-lesion. In unilateral 6-OHDA lesioned rats, SKF 81297, at a dose of 0.5 mg/kg has previously been found to induce striatal cFos expression only in the lesioned hemisphere, reflecting increased D1R sensitivity^23^. Accordingly, we treated 6-OHDA lesioned and control rats with SKF 81297 (0.5 mg/kg, s.c.) and observed an increase in FRET only in lesioned rats (Fig. 3d–f), indicating that D1R-mediated cAMP signalling was enhanced.

### D1R dependent PKA and ERK1/2 activation in dopamine depleted and dyskinetic rats

Long-term treatment with l-DOPA in PD patients frequently leads to the emergence of acute l-DOPA-induced dyskinesia (LID), a debilitating adverse drug effect characterized by abnormal involuntary movements^41,42^. In animal models of PD, dyskinesia can also be induced by D1R agonists^43,44^ or optogenetic activation of dMSNs^45–47^ and the development of LID is dependent on D1R activation^48^ and signalling through PKA and ERK1/2^49,50^.

**Figure 4:**
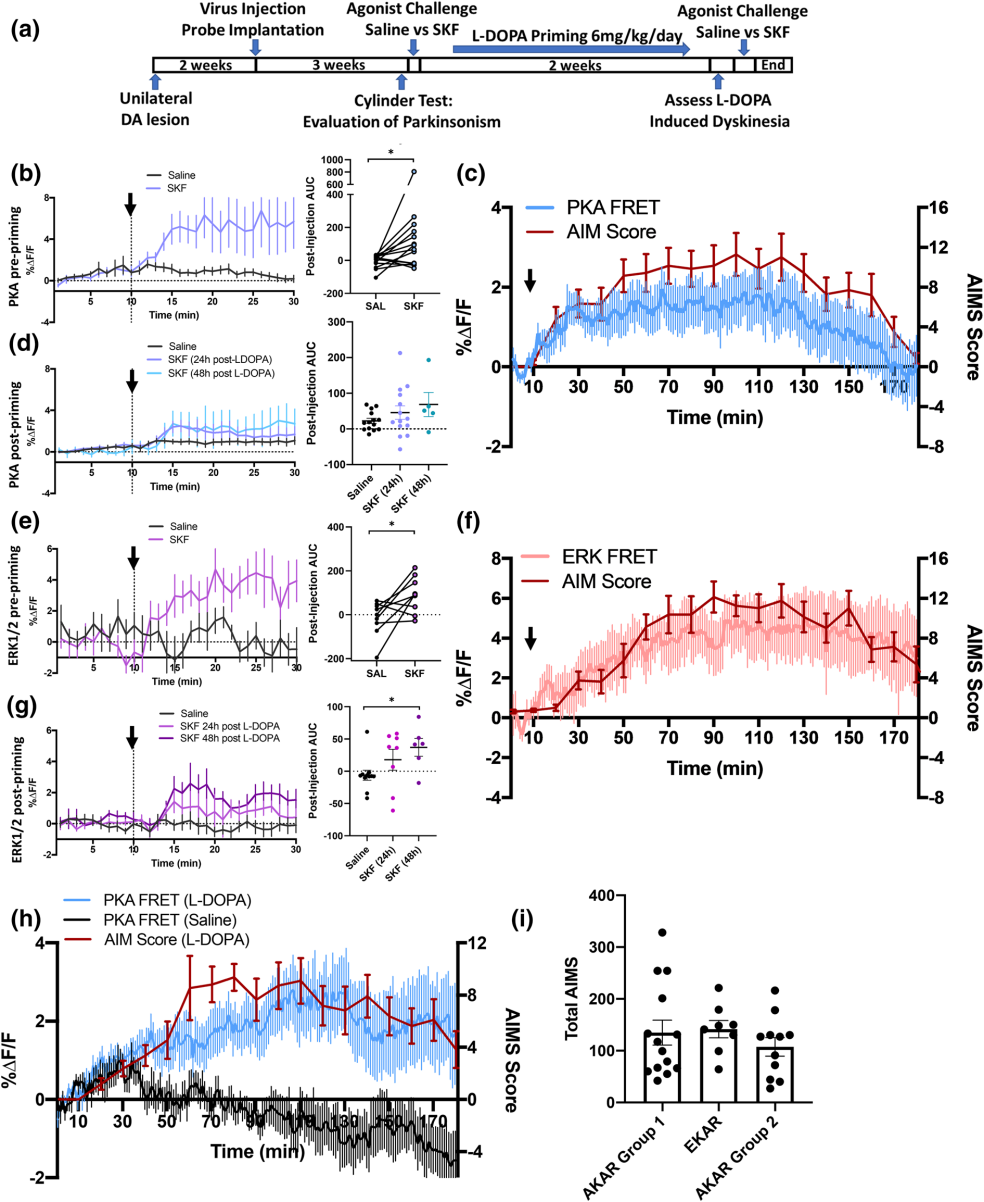
PKA and ERK1/2 signalling in Parkinsonian rats and following development of l-DOPA-induced dyskinesia. (**a**) Timeline of surgeries, drug treatments and testing for l-DOPA-induced dyskinesia (LID) experiments. (**b**) PKA biosensor responses to saline or SKF 81297 (0.5 mg/kg s.c) in drug naive 6-OHDA lesioned rats (n = 14). (**c**) l-DOPA induced dyskinesia and PKA activation measured in 6-OHDA lesioned rats following 2 weeks of l-DOPA priming. l-DOPA/benserazide (6/15 mg/kg s.c.) was injected 10 min after the start of recording and dyskinesia was measured as an AIM score every 10 min (red line), while PKA activation was simultaneously recorded at 2 min intervals (blue line) (n = 14). (**d**) PKA biosensor responses to saline or SKF 81297 in l-DOPA primed, dyskinetic rats, either 24 or 48 h after the last l-DOPA treatment (n = 5, 14). (**e**) ERK1/2 biosensor responses to saline or SKF 81297 in drug naive 6-OHDA lesioned rats (n = 8). (**f**) l-DOPA induced dyskinesia and ERK1/2 activation measured in 6-OHDA lesioned rats following 2 weeks of l-DOPA priming. l-DOPA/benserazide (6/15 mg/kg s.c.) was injected 10 min after the start of recording and dyskinesia was measured as an AIM score every 10 min (red line), while ERK1/2 activation was simultaneously recorded at 2 min intervals (pink line) (n = 7 rats). (**g**) ERK1/2 biosensor responses to saline or SKF 81297 in l-DOPA primed, dyskinetic rats, either 24 or 48 h after the last l-DOPA treatment (n = 6–8 rats). (**h**) A separate group of AKAR expressing rats was prepared in the same manner as those used in (**b**–**d**). After 2 weeks of l-DOPA priming, rats were challenged with saline and l-DOPA/benserazide (6/15 mg/kg s.c.) in a counterbalanced design. Injections occurred 10 min after the start of recording and dyskinesia was measured as an AIM score every 10 min (red line, indicating AIMS in response to l-DOPA), while PKA activation was simultaneously recorded at 2 min intervals (blue and black lines indicate response to l-DOPA and saline respectively). In response to saline injection, rats do not exhibit any detectable AIMS (score of zero) and is not shown (n = 11 rats). (**i**) Total AIM scores induced by post-priming l-DOPA challenge in all groups of rats, as measured as the total AIMS across the 180 min testing period. Data in all time-course plots are mean ± SEM. For (**b**), (**d**), (**e**) and (**f**), the left graph shows time-course of FRET responses, while the right panel shows the integrated area-under the curve (AUC) for the post-injection period. All animals were tested under both saline and drug conditions, as indicated by the connecting lines on the AUC plot. Statistical analysis in (**b**) and (**e**) was performed by paired t-test. Statistical analysis in (**d**) and (**g**) was performed by paired t-test with Bonferroni correction, *p < 0.05 compared to saline.

We first validated that like AAV-AKAR, AAV-EKAR was expressed in cultured striatal neurons and adult rat striatum (Supplementary Fig. S7). We then used fiber-photometry to measure D1R agonist-induced activation of PKA and ERK1/2 in rats that had been dopamine-depleted. We then compared this to the activation of the same pathways following 2 weeks of “priming” with daily l-DOPA injections in a regimen that has previously been shown to produce dyskinesia in 6-OHDA lesioned rats^51,52^ (see Fig. 4a or “Materials and methods” for detailed experimental timeline).

In 6-OHDA lesioned rats recorded before the initiation of l-DOPA treatment, we observed significant activation of striatal PKA by SKF 81297 (0.5 mg/kg s.c.) compared to saline (Fig. 4b). Rats were then treated daily for 2 weeks with l-DOPA (6 mg/kg s.c.) and the DOPA decarboxylase inhibitor benserazide (15 mg/kg s.c.) to induce dyskinesia^51,52^. After 14 days of priming we measured the effect of l-DOPA by recording FRET responses and scoring dyskinesia using the previously described abnormal involuntary movements scoring (AIMS) system^53^. Rats became dyskinetic following l-DOPA injection, and AIM scores peaked within 40 min and returned to baseline 180 min after injection (Fig. 4c); this finding is consistent with previous studies using this model^51,52^. During this acute dyskinesia, PKA activity appeared to be increased, albeit to a lower maximum level than was observed with the earlier SKF 81297 challenge given prior to the start of l-DOPA treatment. We next re-tested the response to SKF 81297 in l-DOPA primed rats after a 24 or 48 h abstinence from l-DOPA and found that after l-DOPA priming, the D1R agonist did not significantly activate PKA compared to saline (Fig. 4d).

Similar results were obtained with the ERK1/2 biosensor, EKAR. In l-DOPA naïve lesioned rats, ERK1/2 was significantly activated by SKF 81297 (0.5 mg/kg, s.c.; Fig. 4e). After l-DOPA priming, EKAR expressing rats also exhibited l-DOPA induced AIMS, and ERK1/2 was also active during dyskinesia (Fig. 4f). Significant activation of ERK1/2 by SKF 81297 was absent 24 h after l-DOPA but restored 48 h after l-DOPA (Fig. 4g).

To rule out the possibility that daily l-DOPA treatments could produce a conditioned response to injection, we tested a separate cohort of rats expressing AKAR and primed with the same regimen consisting of a challenge with SKF 81297 followed by 2-weeks of l-DOPA. Here, we compared the effect of acute l-DOPA to that of saline, on dyskinesia and PKA activation in l-DOPA primed rats (Fig. 4h). As before, acute l-DOPA challenge produced significant dyskinesia, and also increased PKA activity compared to saline injection. The magnitude of l-DOPA induced AIMS was comparable to that seen in the AKAR and EKAR biosensor groups described above (Fig. 4i). In contrast, saline injection alone did not lead to dyskinesia (i.e. zero AIM score; data not shown), and did not activate PKA, suggesting that conditioning had not occurred.

**Figure 5:**
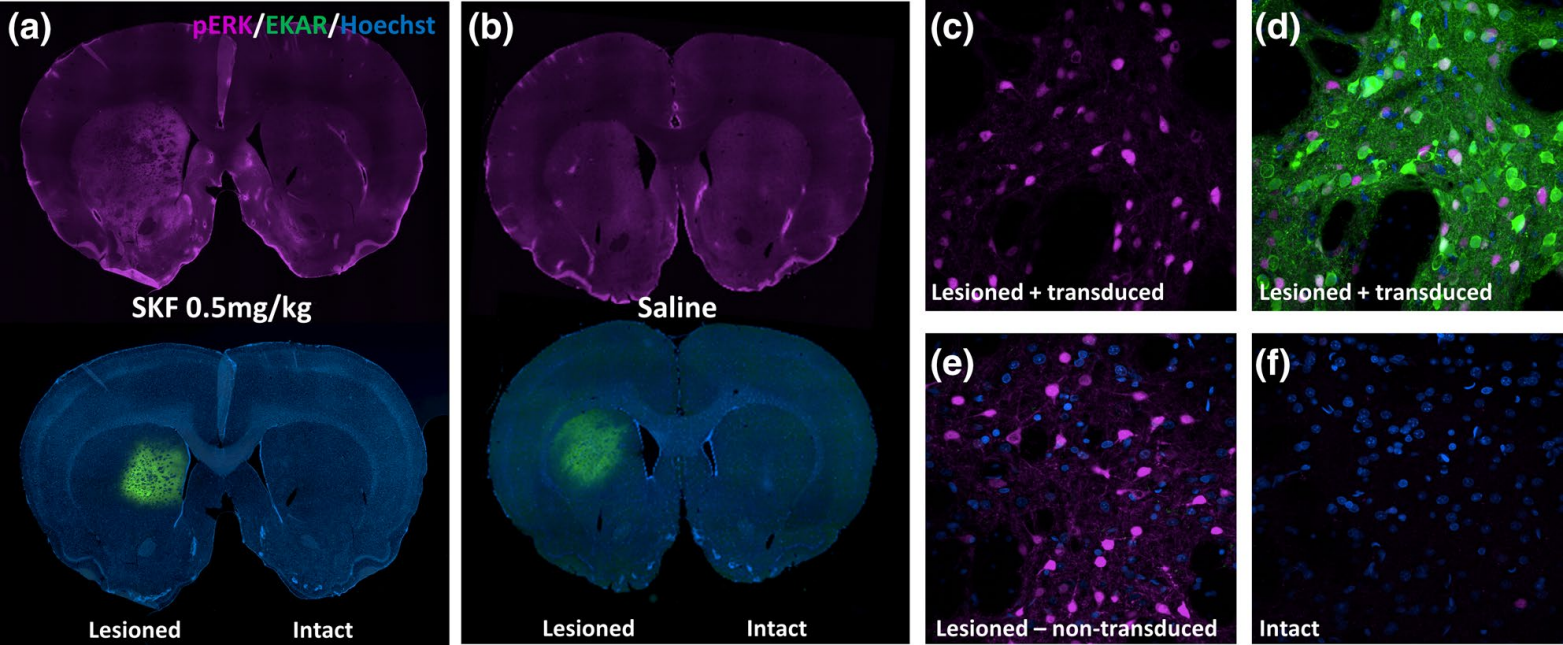
SKF 81297 induced ERK1/2 phosphorylation measured by immunofluorescence in l-DOPA treated, dyskinetic rats. Representative immunofluorescent images depicting phospho-ERK1/2 (pERK) immunolabelling in l-DOPA primed, dyskinetic rats challenged with either (**a**) SKF 81297 (n = 4) or (**b**) saline (n = 3), 24 h following the last l-DOPA treatment. (**c**–**f**) are representative high magnification images of pERK immunolabelling in either transduced (**c**,**d**) or non-transduced (**e**) regions of the lesioned striatum following SKF 81297 challenge, and the non-lesioned striatum following SKF 81297 challenge (**f**).

We next sought to validate our findings by immunolabelling for phosphorylated (active) ERK1/2 in response to challenge with either SKF 81297 or saline (Fig. 5). Following the last photometry recording experiment, rats continued to receive daily l-DOPA for 1 week. Twenty-four hours after the last l-DOPA injection, rats were injected with either saline or SKF 81297 and tissues were fixed 30 min after injection. In saline injected rats, there was no detectable difference in phospho-ERK labelling between the lesioned and intact striata. However, SKF 81297 did induce phospho-ERK selectively in the lesioned hemisphere, an effect that was not detected in our photometry experiment, as discussed above. No difference in ERK phosphorylation levels were observed between virally transduced and non-transduced striatal regions. This discrepancy between results obtained by photometry or immunofluorescence could be the result of the extra week of l-DOPA priming or could indicate a difference between measuring phosphorylation of ERK itself versus measuring ERK protein kinase activity with a biosensor. However, it also suggests that while our technique offers the advantage of tracking changes in signalling in the same animals over time, there is a threshold below which effects were undetected using this photometric approach.

### Repeated l-DOPA enhances dyskinesia but downregulates PKA and ERK1/2 activation induced by D1R agonist

**Figure 6:**
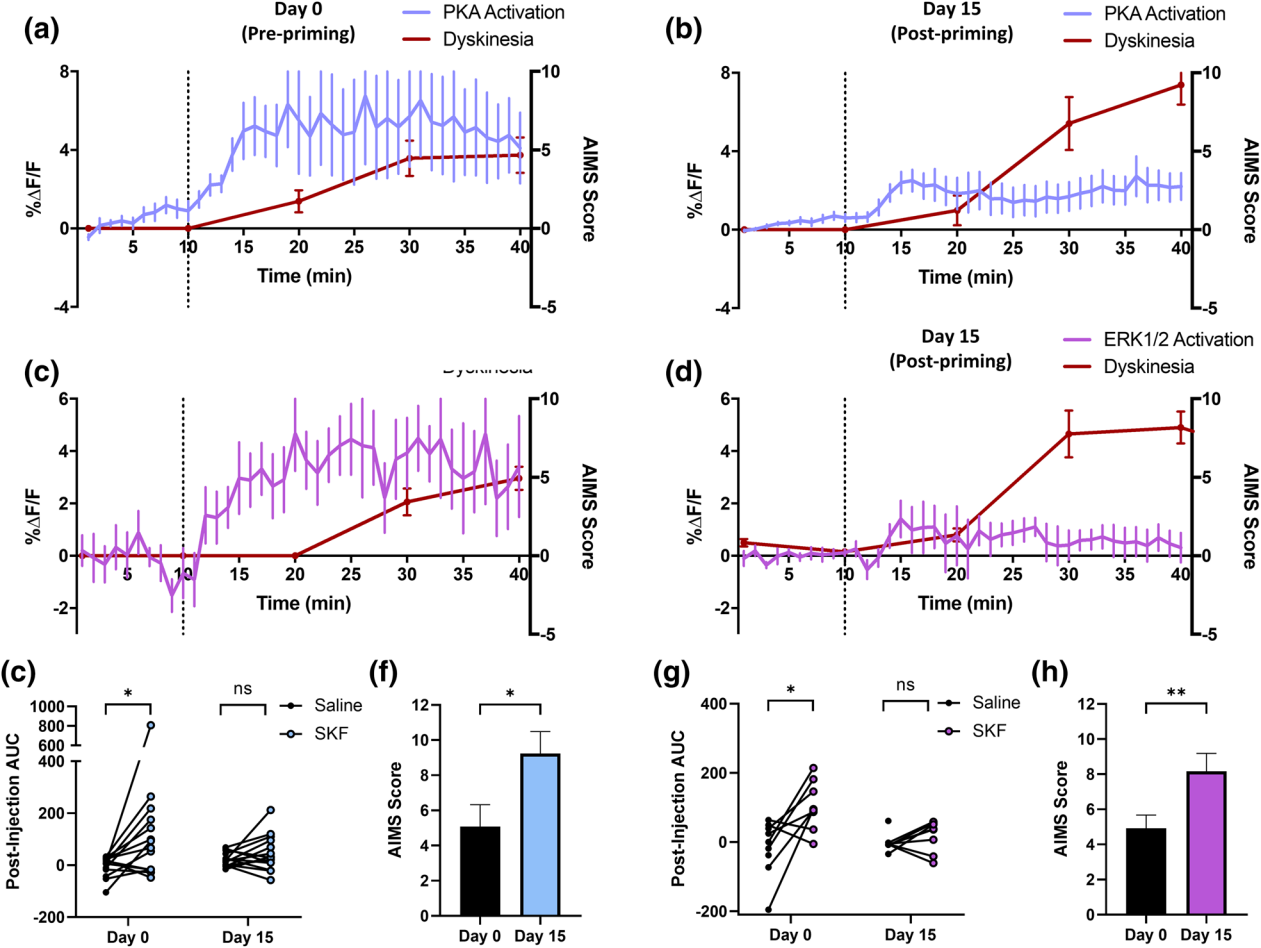
D1R agonist-induced PKA and ERK1/2 signalling is attenuated following chronic l-DOPA, while dyskinesia is significantly enhanced. PKA and ERK1/2 biosensor response profiles (%ΔF/F) and dyskinesia scores (AIMS) in drug naïve, 6-OHDA lesioned rats and l-DOPA primed, dyskinetic rats treated with the D1R agonist SKF 81297 (0.5 mg/kg s.c.) (**a**) PKA biosensor response profiles and dyskinesia scores in drug-naïve, 6-OHDA lesioned rats treated with SKF 81297 (0.5 mg/kg s.c.) (n = 14 rats) (**b**) PKA biosensor response profiles and dyskinesia scores in l-DOPA primed, dyskinetic rats treated with SKF 81297 (n = 14 rats). (**c**) ERK1/2 biosensor response profiles and dyskinesia scores in drug naïve, 6-OHDA lesioned rats treated with SKF 81297 (n = 9 rats) (**d**) ERK1/2 response profile and dyskinesia score in l-DOPA primed, dyskinetic rats treated with 0.5 mg/kg SKF81297 (n = 8 rats). (**e**) Integrated area-under the curve (AUC) for the post-injection period comparing PKA activation by saline versus SKF 81297 before and after l-DOPA priming (**f**) Comparison of peak dyskinesia (measured at 30 min post-injection) in response to SKF 81297 before and after l-DOPA priming (**g**) Integrated area-under the curve (AUC) for the post-injection period comparing ERK1/2 activation by saline versus SKF 81297 before and after l-DOPA priming. (**h**) Comparison of peak dyskinesia (measured at 30 min post-injection) in response to SKF 81297 before and after l-DOPA priming in EKAR rats. All data is presented as mean ± SEM. Statistical analysis of photometry data before and after l-DOPA priming (**e**,**g**) was performed using paired t-test with Bonferroni correction. Comparison of dyskinesia (**f**,**h**) was performed using a Wilcoxon signed-rank test, *p < 0.05, **p < 0.01.

Concurrently with FRET recordings, we compared the behavioral effects of D1R activation in drug naïve and l-DOPA treated animals. In l-DOPA naïve rats, SKF 81297 induced moderate dyskinesia within 30 min of injection, whereas activation of PKA and ERK1/2 occurred as early as 5 min post-injection, and peaked 10–15 min prior to the first AIMs (Fig. 6a,c). After 2 weeks of l-DOPA treatment, the SKF 81297-induced dyskinesia nearly doubled in magnitude, while activation of PKA and ERK1/2 was downregulated (Fig. 6b,d). Using the integrated area under the curve to make statistical comparisons for the signalling data, we confirmed that while PKA and ERK1/2 were significantly activated by SKF 81297 in drug naïve rats, this effect was absent 1 day after the final l-DOPA treatment. In contrast, dyskinesia (measured as the integrated AIM score 30 min after drug injection) was significantly increased (Fig. 6e–h). To determine whether changes in biosensor expression level could have contributed to the reduced effect of SKF 81297 after l-DOPA priming, we compared the fluorescence intensity before and after l-DOPA priming (Supplementary Fig. S8) and found that the expression was either unchanged or slightly increased in all groups, suggesting that the reduced effect could not be explained by biosensor bleaching or loss of expression.

At the completion of the study, tissues were fixed and brain sections were analyzed by immunofluorescence with tyrosine hydroxylase staining to ensure the correct anatomical location of the virus and optical probe and confirm the loss of striatal dopamine terminals (data not shown). Rats that did not show a complete loss of striatal TH immunofluorescence were removed from the analysis. To further validate our LID model we measured two biochemical markers of l-DOPA induced dyskinesia, expression of FosB and phosphorylation of histone 3 on Ser-10 (pS10-H3)^54–56^, and found that both markers were elevated in the lesioned striatum relative to the intact side after l-DOPA priming and SKF 81297 challenge (Supplementary Fig. S9).

## Discussion

To our knowledge, our study is the first to employ ratiometric fiber photometry to study GPCR signalling in live animals. More specifically, we provide a proof-of-concept application of in vivo monitoring of GPCR-dependent protein kinase activity, using ratiometric fiber photometry in combination with FRET-based biosensors of PKA and ERK1/2 activity. This approach revealed D1R-dependent activation of these signalling pathways in freely moving rats. Concordant with previous work, we observed increased activation of PKA and ERK1/2 by D1R in the striatum of 6-OHDA lesioned rats. A chronic regimen of l-DOPA/benserazide that induced dyskinesia also resulted in attenuation of this enhanced signalling, even though D1R-associated dyskinesia was significantly increased. The occurrence of D1R supersensitivity is well established in 6-OHDA lesioned rats (reviewed in^57,58^), and a recent study using FRET-based biosensors has demonstrated hyperactivation of PKA and ERK1/2 by D1R agonists in striatal slices taken from 6-OHDA lesioned mice^59^. The present in vivo study extends these findings, by showing that acute D1R-induced PKA and ERK1/2 activation are downregulated by repeated l-DOPA treatment, suggesting that while D1R signalling may be reduced by chronic l-DOPA treatment, the D1R-driven dyskinesia nevertheless increases.

### Comparison with other approaches

In the past two decades, genetically-encoded FRET biosensors have served as critical tools in revealing spatially and temporally dynamic signalling networks regulated by GPCRs in cultured cells. Many FRET-based sensors have now been created, allowing the optical detection of a wide range of signalling processes including second messengers, ion flux, protein kinases, monomeric GTPases, post-translational modifications and protein–protein interactions^8–11,60^. In contrast, the in vivo application of intracellular signalling-related FRET biosensors is much less developed, especially in freely-moving animals.

Various technologies have been developed in recent years to enable in vivo fluorescence imaging in the brain. Efforts to date have tended to focus on measuring calcium and voltage dynamics with single-fluorophore, intensity-based biosensors. However, FRET-based biosensors are also compatible with a range of imaging technologies, including 2-photon intravital imaging, micro-endoscopy with fibered or head mounted microscopes, and fiber photometry. For example, intravital microscopy, which allows sub-cellular resolution imaging of live cells in native tissue, can be used in transgenic mice expressing FRET biosensors to image signalling dynamics in cell populations throughout the body and brain^2,10,12,13,61^. However, for imaging neurons in conscious animals, the subject needs to be head-fixed to the microscope, an invasive procedure that greatly limits the behavioral repertoire that can be tested. Furthermore, 2-photon imaging approaches are limited to superficial layers of the cortex unless tissue is resected, or a lens is implanted within the brain to reach deeper structures. Epi-fluorescent imaging can be performed on freely moving animals using an endoscope consisting of an implanted lens connected via optical fiber to a conventional microscope, or a head mounted mini-scope. The former approach was previously used to image PKA and ERK1/2 signalling in the nucleus accumbens using transgenic mice expressing the same biosensors used in our study^3^.

Relative to other in vivo fluorescence techniques, the principal advantages of the ratiometric fiber photometry method reported here are simplicity and ease of use. Compared to imaging approaches, photometry occupies a distinct technical niche by sacrificing spatial resolution in favor of reduced cost and invasiveness, a greater range of concomitant behavioral readouts, and the potential for higher throughput or multiplexed recordings from multiple brain regions, which can be accomplished by adding channels to the photometry system^62,63^. Ratiometric photometry requires minimal expertise in optics or image processing, and can be combined in principle with any FRET biosensor in order to study a range of intracellular signalling processes. Furthermore, by using a fiberoptic rotary joint to allow free rotation of the animal being recorded, this technique is highly compatible with rotational behaviors that are a feature of the unilateral 6-OHDA lesion model employed in our study.

### Challenges

Potential challenges to consider for recording FRET signals in vivo include possible tissue autofluorescence and the differential scattering of the donor and acceptor wavelengths of light as they traverse biological tissue. In our hands, the main drawback of this technique was its limited ability to detect small changes in signalling. As described in Fig. 5, we found that an increase in phospho-ERK that was detectable by immunofluorescence was not reported by photometry recording with the EKAR sensor. This could represent a limitation in the sensitivity of either the biosensor, or the photometry-recording. Alternatively, however, it could reflect differences in measuring abundance of pERK by immunofluorescence (where signals are amplified) versus measuring net ERK1/2 protein kinase activity by FRET averaged across all transduced cells in proximity to the optic probe, and takes into account opposing phosphatase activity. Limitations in sensitivity can be addressed by designing more sensitive biosensors with a larger dynamic range. Indeed, a more sensitive AKAR biosensor was recently developed by adding a subcellular localization signal to target the biosensor to PKA-rich locations within the cell^14^. Another limitation of our study was that biosensor expression, although neuron-specific, was not restricted to the neuronal population responsive to D1 agonists, i.e. D1R-expressing neurons. Since the latter represent less than 50% of all striatal neurons, the signal-to-noise ratio could likely be improved through more targeted expression, for example using an appropriate line of Cre-driver transgenic animals.

An additional challenge is the potential for hemodynamic artifacts to affect biosensor outputs. Notably, in a small number of animals, there was a significant *reduction* in fluorescence intensity detected in both CFP and YFP channels after administration of the D1R agonist. Given that D1R agonists can impact blood flow^64–66^ we hypothesized that this effect was caused by alterations in local hemodynamics around the optical probe. Interference from local hemodynamic changes in fluorescent imaging has been previously reported both with FRET-based and single fluorophore, intensity-based biosensors, and experimental and computational approaches have been proposed to address this confounding factor^60,67,68^. In our experiments, the decrease in fluorescence signal caused by the presumed hemodynamic changes led to a reduction in FRET ratios, which could mask the effects of D1R activation. Future studies will be required to explore solutions to each of these challenges.

FRET can be measured both ratiometrically, as in our study, and as a function of fluorescence lifetime. The latter approach requires recording only from the donor moieity of a FRET pair, and because the lifetime of a fluorophore is an intrinsic property of the molecule, lifetime-based measurements are more quantitative compared to ratiometric FRET^69^. Measuring FRET by fluorescence lifetime could circumvent some of the challenges described above. Photometry systems based on time-correlated single photon counting have previously been described^32^ and it was recently shown that this approach could be used to measure PKA activity in the nucleus accumbens using a FRET biosensor optimized for lifetime-based detection^16,70^. As for the imaging techniques described previously, the main drawback of lifetime approaches are the increased equipment costs and complexity of the technique.

### Mechanisms underlying l-DOPA induced dyskinesia

Alterations in D1R signalling play a central role in the development of LID, which is causally dependent on D1R activation and signalling^45–48^. In animal models of PD based on dopamine depletion, D1R signalling becomes more responsive to agonist stimulation, resulting in increased activation of PKA and ERK1/2. Many factors likely contribute to this sensitization including altered D1R surface expression and trafficking, increased expression of Gα_olf_, loss of negative regulatory mechanisms, and abnormal receptor heteromerization^21–23,25,39,71–73^.

Previous studies have demonstrated the importance of PKA and ERK1/2 in the development of dyskinesia. For example, in 6-OHDA lesioned rats, intra-striatal pharmacological inhibition of PKA or ERK1/2 during l-DOPA treatment can attenuate both LID and its molecular correlates such as ΔFosB accumulation^49,50,74^. These studies demonstrate that inhibition of PKA or ERK1/2 during the l-DOPA priming period effectively reduced LID but the role of these pathways in the maintenance or expression of LID, once established, has been less studied. The results of one study indicated that inhibition of ERK1/2 during priming, but not on the day of dyskinesia testing could attenuate LID^50^, suggesting that the primary role of ERK1/2 signaling may be in the development, rather than the expression of LID. Conversely, a study in non-human primates found that established dyskinesia could be reversed by chronic inhibition of striatal ERK1/2 signaling using virally expressed dominant negative mutants of Ras-GRF and ERK2^75^, indicating that ERK1/2 signaling does play an important role in maintaining established LID.

The present findings suggest that while D1R coupling to PKA and ERK1/2 is increased in dopamine depleted rats, the ability of D1R to activate these pathways is attenuated following 2 weeks of l-DOPA treatment. This desensitization of PKA and ERK1/2 activation nevertheless co-occurs with increased coupling of D1R activation to the induction of dyskinesia. Studies using traditional biochemical methods have repeatedly found that D1R dependent ERK1/2 signaling is potentiated in dopamine depleted animals, but the effect of chronic l-DOPA treatment on this upregulated ERK1/2 signaling appears to be more nuanced. Some studies have found that D1R dependent ERK1/2 activation is sensitized in dopamine depleted animals, and that repeated l-DOPA treatment further potentiates ERK1/2 activation to a degree that is correlated with dyskinesia severity^55,76^. On the other hand, others have reported that the sensitized ERK1/2 activation observed in dopamine depleted animals may be reduced after chronic l-DOPA treatment ^21,50,77,78^ and that ERK1/2 attenuation is associated with reduced dyskinesia severity^50,78^. Factors such as lesion severity and the specific l-DOPA regimen may contribute to the different effects of chronic l-DOPA on D1R dependent ERK1/2 activation. Notably, our study represents the first in which activation of these pathways could be measured in the same animals before and after -DOPA priming.

The increased coupling of D1R activation to LID, despite desensitization of these signalling pathways, may reflect PKA- and ERK1/2-dependent remodelling of the striatal direct pathway which occurs at the level of gene expression and synaptic function (reviewed in^58^). Our in vivo observations support a model in which activation of PKA and ERK1/2 during initial exposures to l-DOPA prime dMSNs to become hyper-reactive to subsequent stimulation^47^. This persistent hyperactivity of dMSNs in LID may result from aberrant corticostriatal plasticity regulated by D1R and PKA at the level of the synapse^79–81^ as well as D1R dependent regulation of gene expression, exemplified by the ERK1/2-dependent upregulation of the AP-1 transcription factor subunit ΔFosB, which appears to have a causal role in LID^54,82–84^. These data support a model in which hyperactivation of PKA and ERK1/2 in the dopamine-depleted striatum leads to persistent dyskinesia, despite the fact that these pathways become desensitized in dyskinetic animals.

## Conclusion

We have presented a novel method of ratiometric fiber photometry for monitoring intracellular signalling in freely moving animals using FRET-based biosensors. The system described here could be paired in principle with any FRET biosensor. We have applied this approach to measure sensitization of D1R-induced PKA and ERK1/2 signalling in the striatum following a period of dopamine depletion. The technique further allowed us to demonstrate that l-DOPA treatment partially reverses the sensitization of these signalling pathways, but nevertheless results in greater coupling of D1R activation to the induction of acute dyskinetic symptoms.

## Materials and methods

### Animals

Adult male Sprague–Dawley rats and Sprague–Dawley dams with postnatal day 0 pups were purchased from Charles River, Saint-Constant QC, Canada. Animals were maintained on a 12/12-h light/dark cycle and free access to food and water. All procedures were approved by the McGill University Animal Care Committee, in accordance with Canadian Council on Animal Care Guidelines.

### Drugs

For all in vitro experiments, SKF 81297 HBr (Toronto Research Chemicals), *N*-methyl-d-aspartate (NMDA, Sigma-Aldrich), forskolin (Sigma-Aldrich), SCH 23390 HCl (Tocris) and U0126 (Tocris), were initially dissolved in DMSO to prepare stock solutions and then diluted to the desired concentration in artificial cerebrospinal fluid. For in vivo experiments, SKF 81297 HBr, quinpirole HCl and SCH 23390 HCl (Tocris) were dissolved in physiological saline and injected in a volume of 1 ml/kg. For 6-OHDA lesion experiments, desipramine HCl (Sigma-Aldrich), and pargyline (Sigma-Aldrich) were dissolved in physiological saline and injected at a volume of 1 ml/kg. 6-OHDA HBr (MilliporeSigma) was prepared at 7 µg/µl dissolved in 0.02% ascorbic acid and 0.9% NaCl, and stored at – 80 °C for up to 1 week prior to use. For dyskinesia experiments, l-DOPA methyl ester HCL and benserazide (Sigma) were dissolved in 0.9% saline with 0.1% ascorbate and injected s.c. at 1 ml/kg.

### Cloning and virus production

The FRET-based protein kinase sensors AKAR3EV-NES/NLS and EKAREV-NES/NLS were generously provided by Dr. Michiyuki Matsuda^9^. The pAAV-SynTetOFF plasmid used for all adeno-associated viral constructs was a generous gift from Dr. Hiroyuki Hioki^27^. Subcloning was performed by insertion of a custom multicloning site into the pAAV-SynTetOFF plasmid followed by restriction digestion subcloning to insert each of the biosensors into the AAV backbone.

For cell culture experiments and preliminary in vivo validation (Figs. 1 and 2), adeno-associated virus production, purification and concentration was performed as previously described^85^. Briefly, HEK 293T cells were cultured in DMEM containing 10% fetal bovine serum and 1% penicillin/streptomycin until they were 80% confluent. Calcium phosphate was used to co-transfect cells with pAAV transfer plasmid, pXX680 adenoviral helper plasmid and pXR1 packaging plasmid in a 1:3:1 molar ratio. Cells were harvested 60 h post-transfection and lysed by 3 cycles of freeze–thaw in AAV lysis buffer. Crude AAV preparation was purified by ultracentrifugation on an OptiPrep density gradient and concentrated in PBS using Amicon Ultra centrifugal filters. To determine the AAV titer, viral DNA was extracted using a phenol–chloroform extraction and quantified by qPCR using primers targeted at the viral WPRE sequence. For a detailed protocol, refer to^85^. For experiments involving 6-OHDA lesioned animals (Figs. 3, 4, 5, 6), all viruses were produced by the Neurophotonics Platform Viral Vector Core at Laval University.

### Primary neuronal culture and AAV transduction

Primary striatal neurons were prepared from freshly dissected Sprague–Dawley rat pups at postnatal day 0. The day before dissection, 96-well optical bottom imaging plates (Nunc) were coated with 0.1 mg/ml poly-d-lysine overnight. All solutions were prepared and stored at 4 °C. Prior to dissection, plates were washed three times with sterile water and allowed to dry, and solutions were warmed to 37 °C. The rat pups were decapitated on ice, and brains were rapidly removed and placed in ice cold Hank’s balanced salt solution (HBSS) without calcium and magnesium (Wisent). Striata were dissected and digested for 18 min while rotating at 37 °C with papain (Sigma) diluted to a final concentration of 20 units/ml in a neuronal medium (Hibernate A Minus Calcium, BrainBits). Tissue was then transferred to HBSS containing 10% fetal bovine serum, 12 mM MgSO_4_, and 10 units/ml DNase1 (Roche) for 2 min to halt digestion, and then triturated in HBSS with 12 mM MgSO_4_, and 10 units/ml DNase1 using a fire-polished Pasteur pipette until a cell suspension was produced. The cell suspension was passed through a 40 µm mesh filter (Fisher) to remove undigested tissue and then centrifuged on an OptiPrep (Sigma) gradient as previously described^86^ to remove cell debris and non-neuronal cells such as microglia. Purified neurons were then diluted in Neurobasal-A Medium (NBA) with 1× final concentration of B27 supplement (Gibco), 1% GlutaMAX (Gibco) and 1% penicillin/streptomycin (henceforth referred to as complete NBA) supplemented with 10% fetal bovine serum, and 50,000 cells were plated in a total volume of 75 µl per well. Sixteen hours after plating, cells were washed with warm HBSS and media was changed for complete NBA containing 5 µM cytosine-d-arabinoside to inhibit glial cell growth. Cultures were maintained in complete NBA, and media was refreshed by exchanging 30% of the volume with fresh media every 3 days. Primary striatal neurons were transduced by adding AAV directly into the culture media 3 days after cell plating, using a multiplicity of infection of 5,000 viral genomes/cell. Neurons were then cultured for 7–10 days prior to imaging.

### FRET imaging of primary cultures

One hour prior to imaging, media was exchanged for artificial cerebrospinal fluid (ACSF) as previously described^87^ and cells were returned to the incubator. Imaging was performed at 37 °C and 3% CO_2_ using an Opera Phenix high-content confocal microscopy system (Perkin Elmer). Images were acquired at 40× magnification using a 425 nm laser for excitation of CFP and emissions detected at 435–515 nm (CFP) and 500–550 nm (YFP). All drugs were initially dissolved in DMSO and then diluted 1:1,000 in ACSF. After baseline images were acquired, drug treatment was performed by adding the diluted drugs 1:10 to achieve the final concentration in the well. All image analysis was performed using Columbus analysis software (Perkin Elmer). Briefly, transduced cells were automatically identified based on CFP fluorescence intensity, and then filtered based on morphological properties to exclude non-cell objects. CFP and YFP intensities were determined for each pixel and used to calculate the FRET ratio (YFP/CFP) at each pixel and then the mean FRET for each cell. Between 100 and 2,000 transduced neurons were analyzed per well and used to calculate the mean FRET ratio per well at each time-point. For each well, changes in FRET were expressed as ΔFRET (baseline – stimulated) divided by baseline FRET, and expressed as a percentage (%ΔF/F). Each experiment was performed with at least 2 technical replicates (i.e. 2 wells on the plate receiving the same treatments).

### Stereotaxic surgery

Male Sprague–Dawley rats (Charles River) weighing 275–300 g were anesthetized with isoflurane (3% induction, 1% maintenance) mixed with oxygen, mounted in a stereotaxic apparatus and maintained under isoflurane for the duration of surgery. A midline scalp incision was made and the skull was cleaned in order to visualize bregma and lambda. The angle of the head was adjusted so that the skull was flat and bregma and lambda were at the same height. Using a hand-held micro drill (NeuroStar) with 0.6 mm bit, a hole was drilled for viral injection and probe placement in the dorsal striatum. Additional skull holes were drilled for the insertion of 5 stainless steel surgical screws. Adeno-associated virus (titer ~ 5 × 10^12^ viral genomes/ml) was injected at a volume of 1–2 μl and a rate of 0.2 µl/min using a Hamilton syringe and 26-gauge needle, with the tip located at the following coordinates relative to bregma: + 1.0 AP, + 2.7 ML and − 5.5 DV. For this purpose, a NeuroStar microinjection robot was mounted to the stereotaxic frame. After injection, the syringe needle was left in place for 10 min and then slowly removed. An optic probe (Doric Lens MFC_400/430–0.48_5mm_MF2.5_FLT, 0.4 mm external diameter) was then implanted, with the tip positioned 0.5 mm dorsal to the virus injection site. Once the optical probe was in place, dental cement was used to secure it to the surgical screws. Rats received postoperative monitoring and were administered carprofen analgesic (10 mg/kg s.c.) during surgery and every 24 h for 3 days after surgery. Subsequent testing was performed a minimum of 2–6 weeks post-surgery, to allow expression of the biosensor.

For 6-OHDA lesion experiments, stereotaxic surgery was performed in the same manner, except as follows. Thirty minutes prior to surgery, rats received a subcutaneous injection of pargyline (5 mg/kg) in order to inhibit 6-OHDA degradation, and desipramine HCl (10 mg/kg) to inhibit 6-OHDA uptake into noradrenergic terminals^88^. An injection of 6-OHDA was made in the right medial forebrain bundle (MFB) at the following coordinates relative to bregma: − 2.8 AP, 2.0 ML, − 9.0 DV. Each rat received 2.5 µl of 6-OHDA HBr (7 µg/µl in 0.02% ascorbic acid dissolved in 0.9% NaCl; MilliporeSigma). 6-OHDA was injected at a rate of 0.5 µl/min, with the needle withdrawn after a 10-min delay. The wound was closed with surgical sutures. Animals were allowed to recover for 2 weeks before behavioral evaluation of Parkinsonism.

### Assessment of Parkinsonism

Parkinsonian signs were assessed in the cylinder test, as previously described^88^. Rats were placed individually in a transparent glass cylinder (14 cm diameter × 28 cm height), and observed for 5 min. During this time, an observer noted the number of weight-bearing wall contacts made with each forelimb during rearing, and a video was recorded for later behavioral analysis. Only animals exhibiting preferential use of the ipsilateral forepaw (i.e. in ≥ 70% of rears) were selected to undergo the AAV stereotaxic injections within 1 week of forelimb testing followed by dyskinesia induction 3 weeks after AAV injection.

### Histology and immunofluorescence

Rats were perfused with 0.1% phosphate-buffered saline (PBS) for 10 min followed by 4% paraformaldehyde solution in PBS, pH 7.4, for 10 more min. The brains were extracted and maintained in 4% paraformaldehyde solution for 24 h at 4 °C and then transferred to 0.1% PBS at 4 °C. Coronal or parasagittal sections (30 µm thick), which included the striatum or substantia nigra, were collected using a vibratome (TPI series 1000) at room temperature (RT). Free-floating sections were first washed three times in PBS and then permeabilized for 10 min with 0.3% Triton-100 in PBS. Antigen retrieval was performed by transferring the sections to 80 °C citrate buffer for 30 min, then allowing them to cool to RT. Sections were washed once with PBS and then blocked overnight at 4 °C with 10% bovine serum albumin (BSA) in PBS. Primary antibodies were incubated overnight at 4 °C in PBS 1% BSA. Alexa-fluor conjugated secondary antibodies (Invitrogen) were incubated for 2 h at RT in PBS containing 5% BSA. Nuclei were stained for 10 min at RT with Hoechst 3334 (H3570, Thermo Fisher Scientific) diluted 1:10,000 in PBS, and sections were washed twice more in PBS. Sections were then mounted on microscope slides using Fluoromount mounting medium (E126367, Invitrogen) and stored at 4 °C until imaged. Mounted sections were imaged on the Opera Phenix High Content microscope (Perkin Elmer) using either 5× or 40× objectives. Images acquired at 5× were stitched using the Harmony software (Perkin Elmer) to generate whole brain section images.

The details of the antibody dilutions and sources are as follows. Primary antibodies were: anti-GFP (1:500; sc-8334) and anti-fos-B (1:1,500; sc-48), both from Santa-Cruz; anti-histone H3 pS10 (pH3) (1:300; ab47297) and anti-dynorphin-A (1:500; ab11134), both from Abcam; anti-tyrosine hydroxylase (1:1,000; P40101), from Pel-Freez. Anti-DARPP-32 (1:1,000, #2302), from Cell Signalling; anti-GFAP (1:1,000; GA524), from Agilent. Secondary antibodies were: anti-mouse Alexa 488 (1:500; A21236) and anti-rabbit Alexa 647 (1:500; A21245), both from Invitrogen.

### Construction of ratiometric photometry recording system

The FRET acquisition system was designed and built by Labeo Technologies (Montréal, QC, Canada). The excitation source is a laser diode centered at 450 nm with an optical filter at 438 ± 12 nm. A dichroic mirror (with a cut-off wavelength of 458 nm) allows separation of excitation and emission channels. On the detection side, two photomultiplier tubes (PMTs, Hamamatsu Photonics) are used to measure fluorescence. Each detection channel is coupled with an emission filter (483 ± 16 nm for CFP and 547 ± 27 nm for YFP) and another dichroic mirror (cut-off wavelength of 520 nm) is used to split them. PMT signals are amplified with trans-impedance amplifiers and acquired with a data acquisition device (National Instruments) at up to 1 kHz, with the possibility to record up to 4 auxiliary analog inputs in synchrony. The acquisition electronics are connected to a computer through a USB port. Both excitation and emission optical pathways are co-aligned and connected to a 400 µm optical fiber which is connected to a 400 µm fiberoptic patch cable (Doric Lens), which can be connected to the animal directly, or with an intervening rotary joint (Doric Lens).

The acquisition user interface is used to control laser power, acquisition frequency and program the duration and frequency for periodic recordings. A Matlab analysis script provided with the FRET acquisition system is used to open saved acquisitions for post-processing and allows visualization of the recordings and generation of basic descriptive statistics. Following processing in Matlab, the data is exported in CSV format for subsequent analysis.

### Photometry recording and analysis

For recordings in anesthetized animals, rats were rapidly anesthetized in an induction chamber using 3% isoflurane and then maintained at 0.5% isoflurane while mounted loosely on a stereotaxic frame with a nose-cone to supply oxygen and isoflurane. Under this level of anesthesia rats were unconscious but maintained normal reflexive responses. The photometry system patch cable was connected to the surgically implanted optic probe using a ceramic mating sleeve (Doric Lens). Baseline recordings of at least 10 min were acquired prior to drug administration. For experiments in freely moving rats, a fiberoptic rotary joint (Doric Lens) was mounted above the test chamber allowing the rats to freely rotate while connected to the photometry patch cable.

All photometry recording was performed using laser power of 20 µW and with PMT gain set to 70% for both the CFP and YFP channels. To minimize bleaching or photo-toxicity, the laser was turned on for only 30 out of every 60 s (for test sessions < 60 min long) or 30 out of every 120 s (for longer test sessions). Data was initially processed using a custom MatLab script to stitch together individual 30-s recordings and downsample the data from 1,000 to 100 Hz for subsequent analysis.

Data from each AKAR and EKAR recording were analyzed using a custom R script we wrote to perform the following series of operations. The first step was to subtract background autofluorescence which was found to decrease over time in response to sustained illumination. Accordingly, age-matched control (sham-injected) rats were used to calculate background CFP and YFP values; these control rats were recorded under identical conditions to experimental subjects. The mean background for CFP and YFP was plotted as a function of time (Fig. 2, Supplementary Fig. S6) and fit to a linear equation which was then used to subtract CFP and YFP background at each time-point. After background subtraction, the FRET ratio was calculated as CFP/YFP. Baseline FRET was calculated as the mean FRET for the pre-injection period and all measurements were subsequently expressed as the percentage ΔFRET relative to baseline FRET [i.e. %ΔF/F = 100 * (FRET − FRET_baseline_)/ FRET_baseline_]. The mean %ΔF/F for each experimental group were then plotted. Area under the curve was calculated from the %ΔF/F *vs.* time plots using the following formula, where *C* is the %ΔF/F value corresponding to each timepoint (*T*), and B is defined as the mean %ΔF/F of the baseline period.

$$AUC = 0.5i = \ln - 1\left( {Ti + l + Ti} \right)\left( {Ci + l + Ci - 2B} \right)$$

### Drug treatments during photometry recording

Drug treatments of saline versus agonist in photometry experiments were generally performed in a counterbalanced, repeated measures design with each animal being tested under both saline and drug conditions on consecutive days, with the following exception. In Fig. 1e, the SKF 81297 (10 mg/kg) and SCH 23390 tests were performed on separate days in a non-counterbalanced fashion. Antagonist pre-treatments were administered 30 min prior to the beginning of a test session. All drug injections during photometric recordings were performed during a laser-OFF interval and without disconnecting the animal from the recording system. Experimenters were not blinded to drug conditions.

### Dyskinesia induction and AIM assessment

In order to model l-DOPA induced dyskinesia, Parkinsonian rats were primed to elicit abnormal involuntary movements (AIMs) with once-daily injections of l-DOPA/benserazide (6/15 mg/kg s.c.) for 14 days. AIMs were then assessed the day after the last priming injection. To this end, rats were placed in glass cylinders and AIMs were scored for 2 min at 10-min intervals. l-DOPA/benserazide (6/15 mg/kg) was injected 10 min after rats were placed in the cylinder and each test lasted for a total of 3 h. Orolingual, limb and axial dyskinesias were scored according to the scales as previously described^53^. The integrated AIMs score was then calculated as the sum of the intensity score multiplied by the duration score for each of the behaviors scored (orolingual, limb and axial). The same procedure was used to score AIMS in rats treated with saline or SKF 81297 (0.5 mg/kg). Simultaneous photometry recording was performed during all dyskinesia experiments.

### Experimental design for dyskinesia experiments

There were three main in vivo experiments. The first two of these used the AKAR and EKAR biosensors, respectively. The third experiment used the AKAR biosensor and served to replicate the first experiment, with a slightly modified design as indicated below.

Surgeries for injection of AAV and probe placement was performed 2 weeks after 6-OHDA lesion. Three weeks after this second surgery, rats were assessed for Parkinsonism in the cylinder test, and rats that demonstrated less that 70% preference for the intact forelimb were excluded from the study. Photometry and dyskinesia assessment were then performed with each rat receiving an acute injection of saline and SKF 81297 administered on 2 consecutive days in a counterbalanced design. Rats were then treated daily for 14 days with l-DOPA and benserazide, and photometry was performed and AIMS assessed after the final l-DOPA treatment, as described in the preceding section. The response to saline and SKF 81297 was then re-assessed 24 or 48 h after the last dose of l-DOPA, as indicated. All rats continued to receive daily l-DOPA until 24 h prior to perfusion. Thirty minutes prior to PFA perfusion, rats received either saline or SKF 81297. For these experiments, 14 rats expressing AKAR and 9 rats expressing EKAR were used.

In the third experiment, rats received identical drug treatments as described above, with the exception that after the 14th day l-DOPA priming, the AIMS and photometry were measured after acute administration of both l-DOPA and saline in a counterbalanced design. Eleven rats were used in this experiment.

### Statistical analysis

All statistical testing was performed using Prism 8 (GraphPad). For cell culture experiments, data for each biosensor was analyzed first by 2-way ANOVA, with multiple comparisons performed using Bonferroni-corrected t-tests. Area-under-the-curve analysis of in vivo recording data was performed as follows. For comparison of saline *vs.* SKF 81297 at single time-points (Fig. 4b,c), data were analyzed by paired t-tests. For comparisons of saline *vs.* SKF 81297 at multiple time-points before and after l-DOPA (Figs. 4e,f and 6e,g), multiple comparisons were made by Bonferroni corrected paired t-tests comparing each SKF 81297 condition to saline. Comparison of dyskinesia score before and after l-DOPA treatment was performed by a Wilcoxon signed-rank test (Fig. 6f,h).

## Supplementary information

Supplementary Information.
