# Supplementary material for: Dopamine D1 receptor signalling in dyskinetic Parkinsonian rats revealed by fiber photometry using FRET-based biosensors
Source: Sci Rep. 2020 Sep 2;10:14426. doi: 10.1038/s41598-020-71121-8 (PMC7468292; doi:10.1038/s41598-020-71121-8)
Supplement: Supplementary file 1 — Supplementary Information. [file 41598_2020_71121_MOESM1_ESM.pdf]

Dopamine D1 receptor signalling in dyskinetic Parkinsonian rats revealed by fiber photometry using FRET-based biosensors

Jace Jones-Tabah[1], Hanan Mohammad[1], Shadi Hadj-Youssef[1], Lucy E.H. Kim[1], Ryan D. Martin[1], Faïza Benaliouad[1], Jason C. Tanny[1], Paul B.S. Clarke[1]* and Terence E. Hébert[1]*

[1]Department of Pharmacology and Therapeutics, McGill University, Montréal, Québec, H3G 1Y6, Canada

Keywords: G protein-coupled receptors; biosensors; resonance energy transfer; cellular signalling; fiber photometry; *in vivo* drug discovery

Running title: Photometry recording of FRET-based biosensors in rat brain

* Co-corresponding authors- send correspondence to:

Dr. Terence E. Hébert, PhD,

Department of Pharmacology and Therapeutics,

McGill University,

3655 Promenade Sir-William-Osler, Room 1325

Montréal, Québec, H3G 1Y6, Canada

Tel: (514) 398-1398

E-mail: terence.hebert@mcgill.ca

OR

Dr. Paul Clarke, PhD,

Department of Pharmacology and Therapeutics,

McGill University,

3655 Promenade Sir-William-Osler, Room 1325

Montréal, Québec, H3G 1Y6, Canada

Tel: (514) 398-3616 ext 1

E-mail: paul.clarke@mcgill.ca

Supplementary Figures and Figure Legends

Supplementary Figure S1: AAV_1-SynTet-AKAR expresses in NeuN and DARPP-32 positive cells, but not in GFAP positive cells in primary neuronal culture.

Representative images showing primary neurons transduced with AAV_1-SynTet-AKAR (green) and immunolabelled with either a general neuronal marker, NeuN (panel A), a marker of striatal medium-spiny GABAergic neurons, DARPP-32 (panel B), or an astrocytic marker, GFAP (panel C). White and orange triangles respectively indicate AKAR expressing cells that are positively or negatively labelled for the indicated proteins. Nuclei are labelled with Hoechst stain (blue). Representative images were selected from the set of images acquired by high-content imaging of primary neurons plated on 96-well plates. At least 3 wells were each stained for NeuN, DARPP-32 and GFAP, and more than 25 images per well were acquired.

Supplementary Figure S2: AAV_1-SynTet-AKAR expresses in NeuN and DARPP-32 positive, but not GFAP positive cells in the dorsal striatum

(a-c) Representative images of fixed striatal tissue sections from rats injected with AAV_1-SynTet-AKAR (green) and immunolabelled with a general neuronal marker, NeuN (a), a marker of striatal medium-spiny GABAergic neurons, DARPP-32 (B), or an astrocyte marker, GFAP (c). Nuclei are labelled with Hoechst stain (blue). **(d)** Sagittal section from a rat injected in the dorsal striatum (caudate/putamen) with AAV_1-SynTet-AKAR (green). White lines indicate fluorescently labelled axons projecting from the striatum to the target nuclei of the indirect pathway and direct pathways, the external globus pallidus (GPe) and substantia nigra reticulata (SNr) respectively.

Supplementary Figure S3: AAV_1-SynTet-AKAR expresses dynorphin-positive and dynorphin-negative striatal neurons

Representative images of fixed striatal tissue sections from rats injected with AAV_1-SynTet-AKAR (green) and immunolabelled for dynorphin-A (purple), a neuropeptide expressed in direct pathway neurons. Orange and white arrows indicate transduced neurons that are positively or negatively stained for dynorphin, respectively.

Supplementary Figure S4: FRET biosensor emission spectra

Emission spectra of AKAR expressed in HEK 293 cells under 450 nm excitation. Emission spectra were determined under basal conditions (DMSO vehicle) and following treatment with a saturating concentration of the adenylyl cyclase activator forskolin. Areas shaded in blue and orange represent the spectral range of the emission filters used for CFP and YFP in the photometry system (see *Materials and Methods* for additional details). Fluorescence emission is shown in arbitrary units.

Supplementary Figure S5: Detection of fluorescence *in vivo* in probe-only *vs.* AKAR-injected rats

Intensity of emissions recorded by photometry in the CFP and YFP channels using 450 nm excitation at 20 μW laser power. Recordings were made in either rats with optical probes only or 3 weeks after injection of AAV_1-SynTet-AKAR. Fluorescence intensity is measured as photomultiplier tube output and is displayed as mV units. Each point on the graph represents an individual control or AAV-transduced rat.

Supplementary Figure S6: Acquisition and processing of photometry data

(a) FRET ratio of unprocessed fluorescent data. As described in *Materials and Methods*, data were acquired at 100 Hz in discrete 30-s intervals, with the laser turned off between sampling intervals. **(b)** Time course showing autofluorescence in CFP and YFP channels, recorded in the same way from control rats that were not injected with virus. Data displayed represents the mean ± SEM at each time (n = 2 rats). The dotted line represents a linear fit used to calculate the expected autofluorescence in each channel at a specified time point. **(c)** Flow chart describing the steps of data processing used to calculate the %ΔF/F measure.

Supplementary Figure S7: Validation of AAV-EKAR expression in primary striatal neurons and adult rat striatum.

(a) Representative images showing primary neurons transduced with AAV_1-SynTet-EKAR (green) and immunolabelled with NeuN, DARPP-32 or GFAP. Representative images were selected from the set of images acquired by high-content imaging of primary neurons plated on 96-well plates. At least 3 wells were stained for each of NeuN, DARPP-32 and GFAP, and more than 25 images per well were acquired. **(b)** Representative images of fixed striatal tissue sections from rats injected with AAV_1-SynTet-EKAR (green) and immunolabelled with NeuN, DARPP-32 or GFAP. Nuclei are labelled with Hoechst stain (blue). The same set of neurons is shown within a given row.

Supplementary Figure S8: Comparison of biosensor expression levels before and after L-DOPA priming

Fluorescence intensities and corresponding FRET ratio under basal conditions for each group of rats tested, before and after L-DOPA priming. Fluorescence signals were detected by photometry using a constant 20μW laser power. Fluorescence intensity is measured as photomultiplier tube output and is displayed as mV units. Each point represents an individual AAV-transduced rat.

Supplementary Figure S9: Immunofluorescent staining for markers of dyskinesia in 6-OHDA lesioned, L-DOPA treated rats.

Representative images showing immunolabelling of FosB and serine-10 phosphorylated histone 3 (pSer10-H3) in tissue from 6-OHDA lesioned rats after 2 weeks of daily treatment with L-DOPA/benserazide (6/15 mg/kg s.c.) and acute challenge with SKF 81297 (0.5mg/kg) 30 minutes prior to perfusion (n = 4).

Figure S1

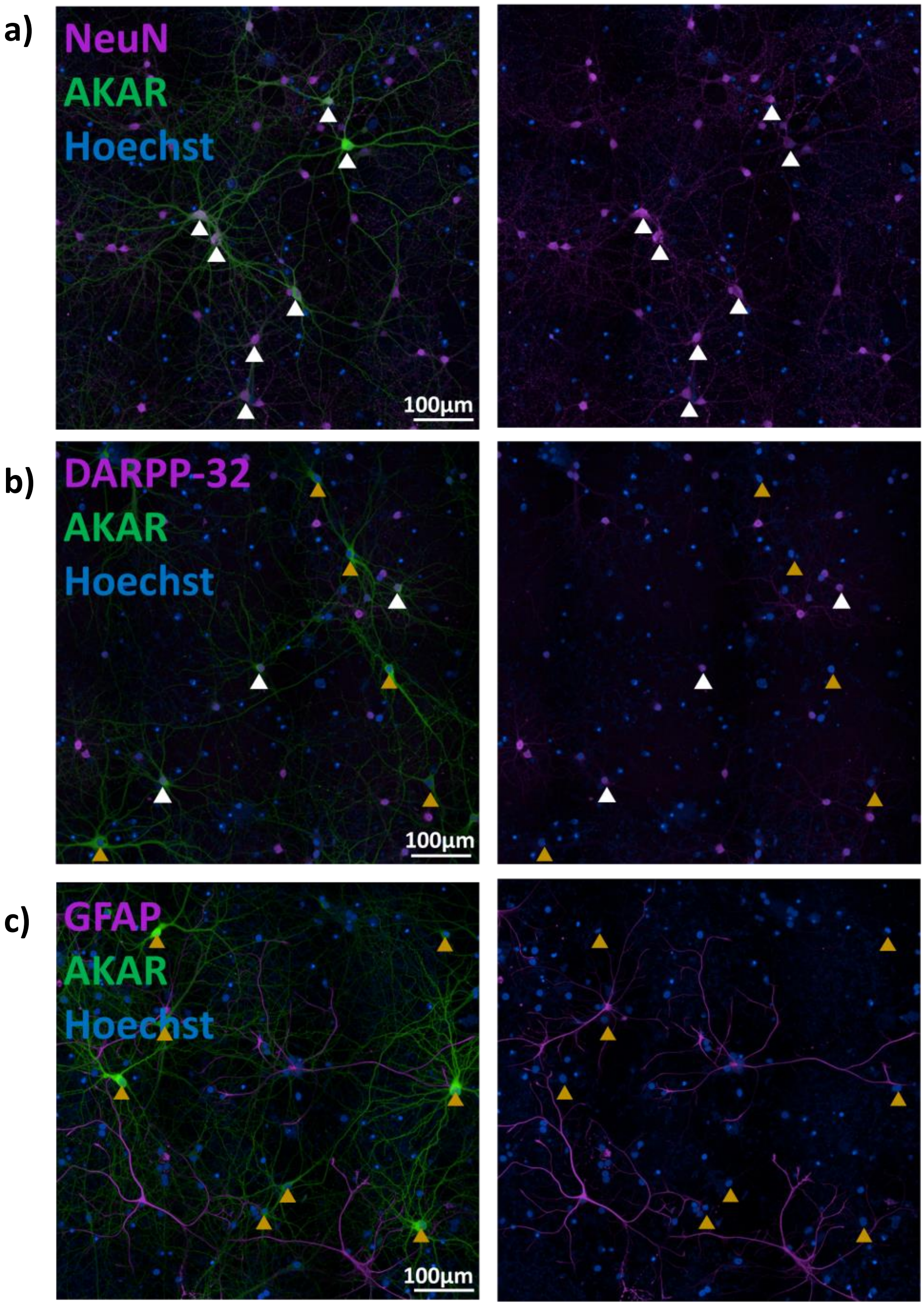
a)
NeuN
AKAR
Hoechst
100µm
b)
DARPP-32
AKAR
Hoechst
100µm
c)
GFAP
AKAR
Hoechst
100µm

Figure S2

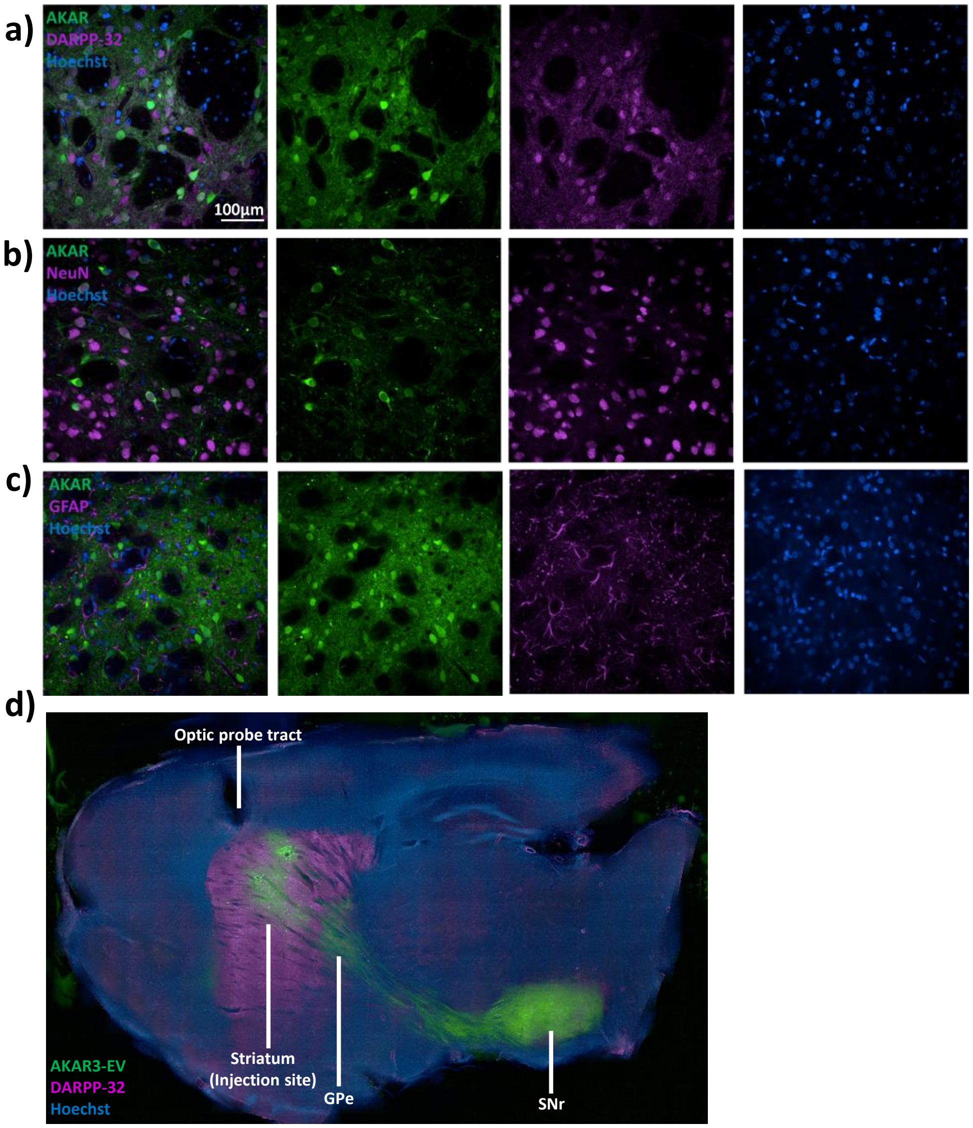
a)
AKAR
DARPP-32
Hoechst
100µm
b)
AKAR
NeuN
Hoechst
c)
AKAR
GFAP
Hoechst
d)
Optic probe tract
Striatum
(Injection site)
GPe
SNr
AKAR3-EV
DARPP-32
Hoechst

Figure S3

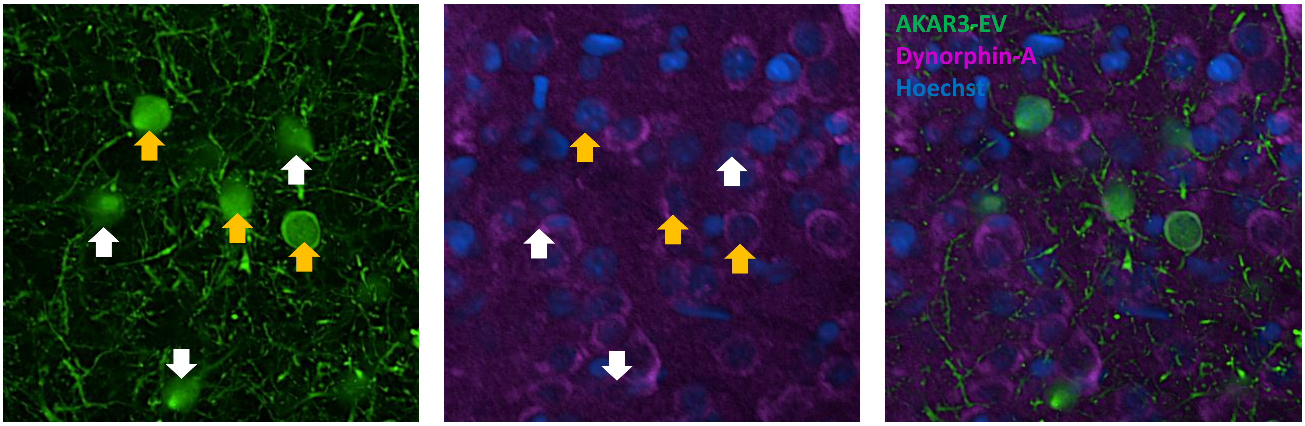

Figure S4

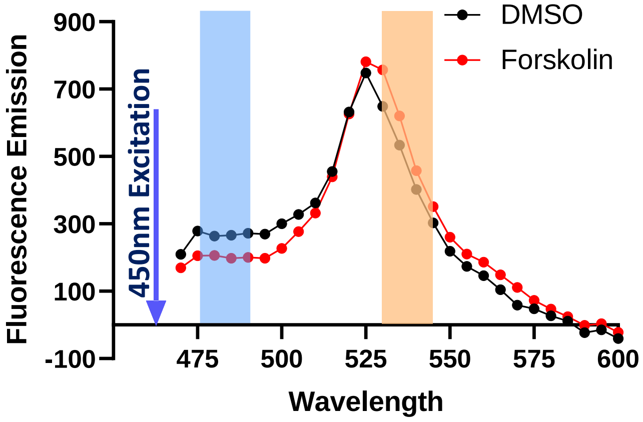

900
700
500
300
100
-100
Fluorescence Emission
450nm Excitation
475
500
525
550
575
600
Wavelength
DMSO
Forskolin

Figure S5

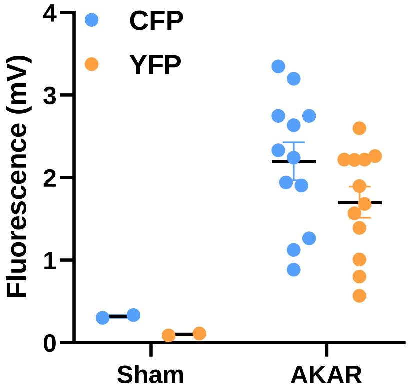

Figure S6

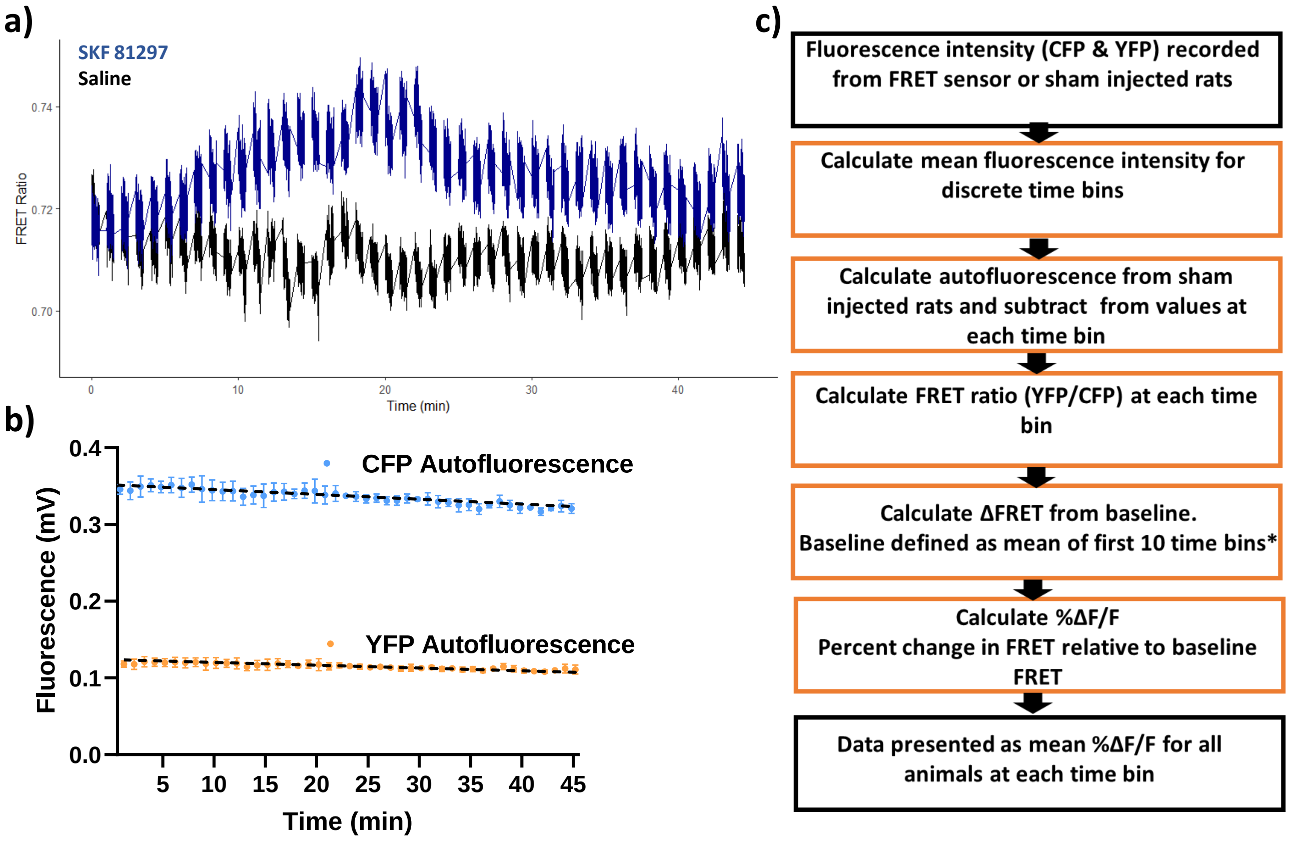
a)
SKF 81297
Saline
FRET Ratio
0.74
0.72
0.70
0
10
20
30
40
Time (min)
b)
CFP Autofluorescence
YFP Autofluorescence
Fluorescence (mV)
0.4
0.3
0.2
0.1
0.0
5
10
15
20
25
30
35
40
45
Time (min)
c)
Fluorescence intensity (CFP & YFP) recorded from FRET sensor or sham injected rats
Calculate mean fluorescence intensity for discrete time bins
Calculate autofluorescence from sham injected rats and subtract from values at each time bin
Calculate FRET ratio (YFP/CFP) at each time bin
Calculate ΔFRET from baseline. Baseline defined as mean of first 10 time bins*
Calculate %ΔF/F Percent change in FRET relative to baseline FRET
Data presented as mean %ΔF/F for all animals at each time bin

Figure S7

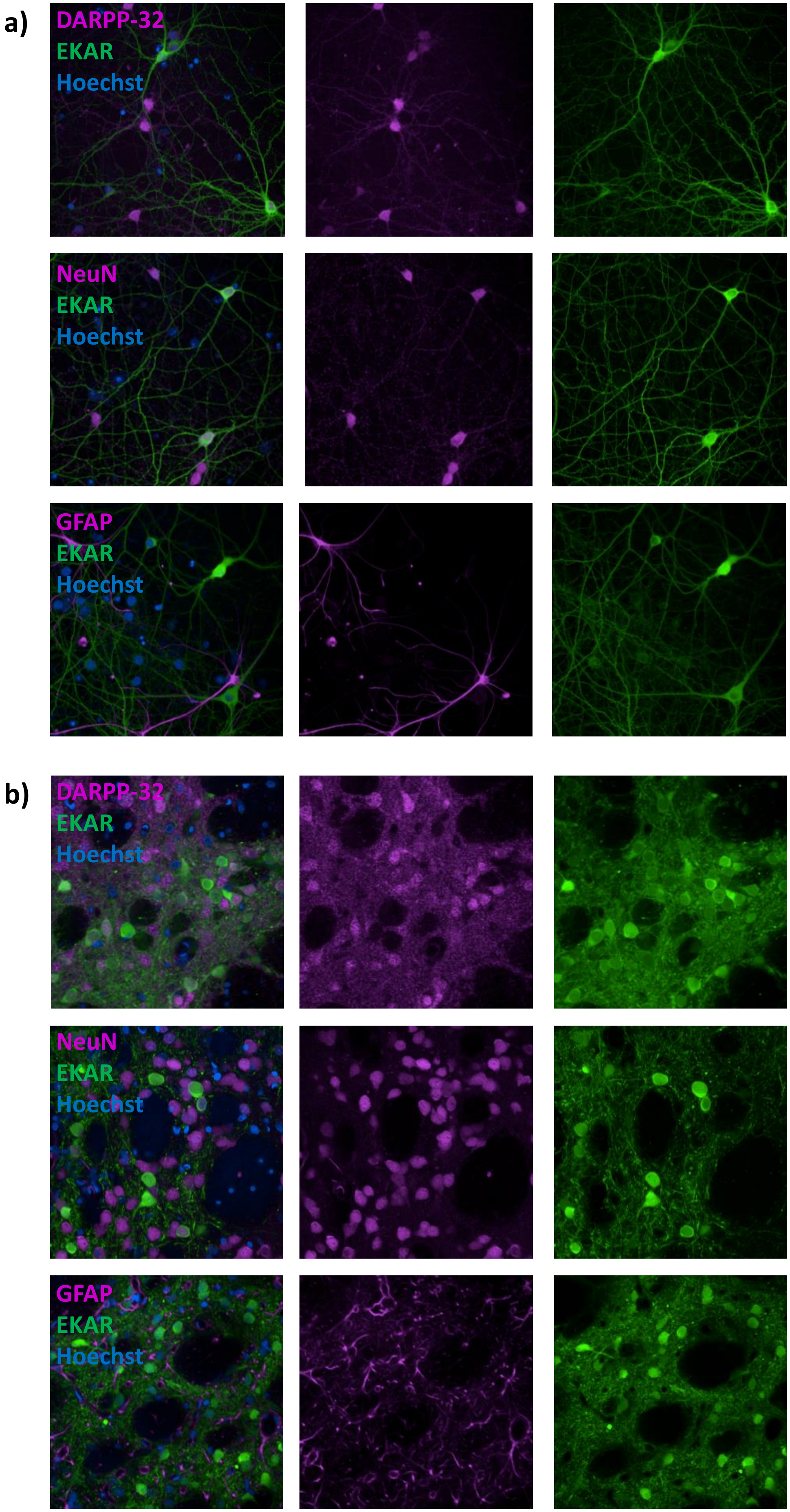
a)
DARPP-32
EKAR
Hoechst
NeuN
EKAR
Hoechst
GFAP
EKAR
Hoechst
b)
DARPP-32
EKAR
Hoechst
NeuN
EKAR
Hoechst
GFAP
EKAR
Hoechst

Figure S8

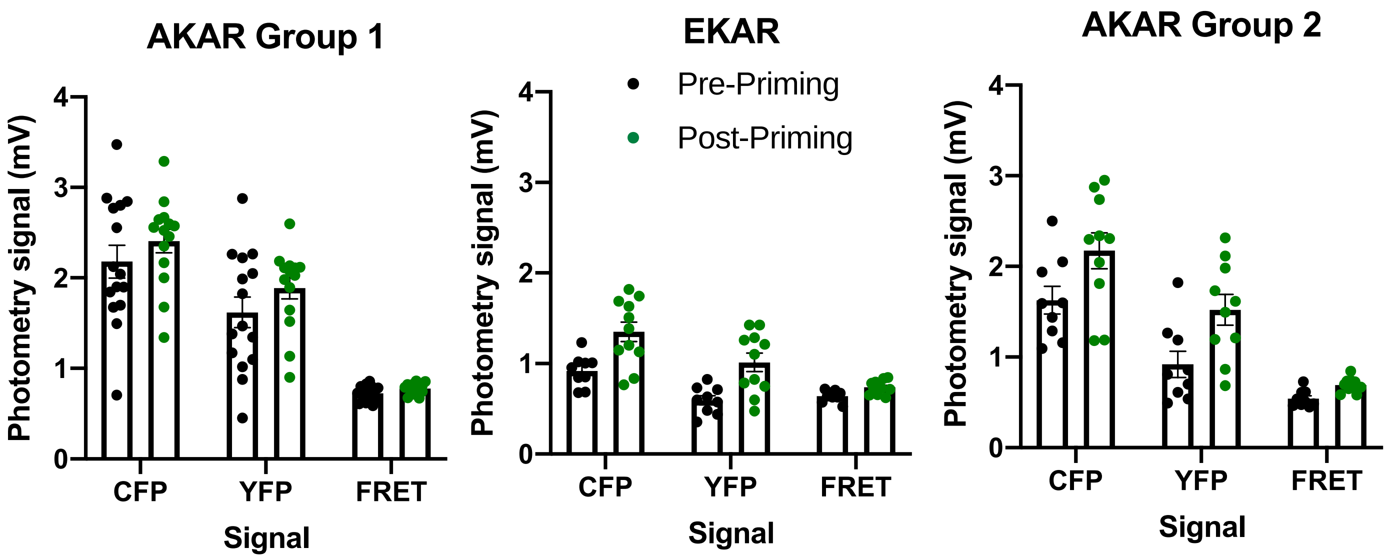

AKAR Group 1
EKAR
AKAR Group 2
Pre-Priming
Post-Priming
Photometry signal (mV)
0
1
2
3
4
CFP
YFP
FRET
Signal

Figure S9

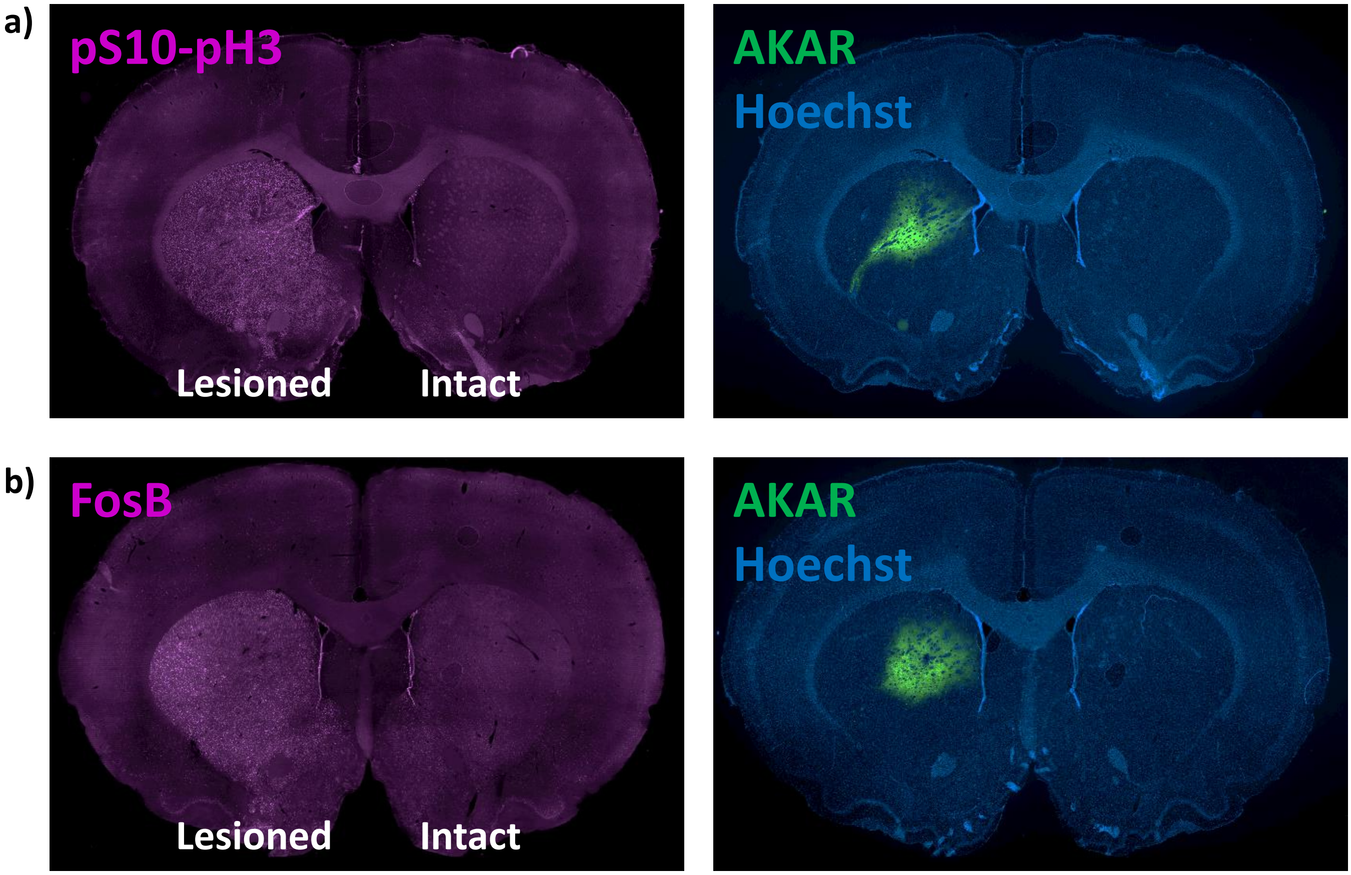
a)
pS10-pH3
Lesioned
Intact
AKAR
Hoechst
b)
FosB
Lesioned
Intact
AKAR
Hoechst
